# TGFβ family signaling in human stem cell self-renewal and differentiation

**DOI:** 10.1186/s13619-024-00207-9

**Published:** 2024-11-28

**Authors:** Sijia Liu, Jiang Ren, Yanmei Hu, Fangfang Zhou, Long Zhang

**Affiliations:** 1 International Biomed-X Research Center, Key Laboratory of Precision Diagnosis and Treatment for Hepatobiliary and Pancreatic Tumor of Zhejiang Province, Second Affiliated Hospital of Zhejiang University School of Medicine, Zhejiang University, Hangzhou, China; 2 The First Affiliated Hospital, MOE Basic Research and Innovation Center for the Targeted Therapeutics of Solid Tumors, Institute of Biomedical Innovation, School of Basic Medical Sciences, Jiangxi Medical College, Nanchang University, Nanchang, China; 3 Department of Endocrinology and Metabolism, The First Affiliated Hospital of Zhengzhou University, Zhengzhou, 450052 China; 4 The First Affiliated Hospital, the Institutes of Biology and Medical Sciences, Suzhou Medical College, Soochow University, Suzhou, China; 5 MOE Key Laboratory of Biosystems Homeostasis & Protection and Innovation Center for Cell Signaling Network, Life Sciences Institute, Zhejiang University, Hangzhou, 310058 China

**Keywords:** TGFβ, BMP, ESC, iPSC, Cancer stem cell

## Abstract

Human stem cells are undifferentiated cells with the capacity for self-renewal and differentiation into distinct cell lineages, playing important role in the development and maintenance of diverse tissues and organs. The microenvironment of stem cell provides crucial factors and components that exert significant influence over the determination of cell fate. Among these factors, cytokines from the transforming growth factor β (TGFβ) superfamily, including TGFβ, bone morphogenic protein (BMP), Activin and Nodal, have been identified as important regulators governing stem cell maintenance and differentiation. In this review, we present a comprehensive overview of the pivotal roles played by TGFβ superfamily signaling in governing human embryonic stem cells, somatic stem cells, induced pluripotent stem cells, and cancer stem cells. Furthermore, we summarize the latest research and advancements of TGFβ family in various cancer stem cells and stem cell-based therapy, discussing their potential clinical applications in cancer therapy and regeneration medicine.

## Background

**Fig. 1:**
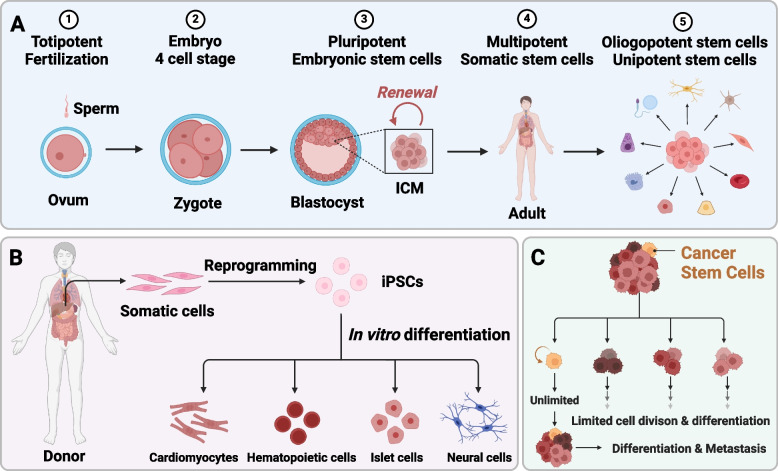
Schematic illustration of different types of human stem cells. **A** Stem cells can be categorized into totipotent, pluripotent, multipotent, oligopotent, and unipotent stem cells based on their varying degrees of differentiation potential. **B** Induced pluripotent stem cells are generated through the reprogramming of somatic cells into a pluripotent state. **C** Cancer stem cells constitute a minor subset of undifferentiated cancer cells that possess characteristics resembling those of stem cells and are responsible for tumor differentiation and metastasis

Human stem cells are present in embryonic, postnatal, and adult tissues (Fuchs and Segre 2000). Based on their differentiation potential, stem cells can be classified into five types: totipotent, pluripotent, multipotent, oligopotent, and unipotent stem cells (Fig. 1A) (Essawy et al. 2020; Apostolou et al. 2023). Totipotent stem cells exhibit the highest capacity for proliferation and differentiation, as demonstrated by the zygote's ability to generate all embryonic and extraembryonic structures (Baker and Pera 2018; Xu et al. 2022). Pluripotent stem cells, such as embryonic stem cells (ESCs), have a lower potency compared to totipotent cells and can differentiate only into various embryonic tissues (Donovan and Gearhart 2001; Du and Wu 2024). Somatic stem cells, also known as adult stem cells or tissue-specific stem cells, are multipotent stem cells. They have the ability to differentiate into cell types specific to their tissue or organ of origin, such as hematopoietic stem cells and neural stem cells (Ferraro et al. 2010; Zakrzewski et al. 2019). Oligopotent stem cells are more restricted in their differentiation potential but can still produce specific cell types within certain tissues, such as the common lymphoid progenitor, which generates T lymphocytes, B lymphocytes, and natural killer cells (Bryder et al. 2006; Warner et al. 2012). Furthermore, unipotent stem cells exhibit the highest degree of lineage restriction, giving rise exclusively to cells within a specific lineage, such as spermatogonial stem cells (de Rooij 2017, Yu et al. 2022).

In addition to the presence of human endogenous stem cells, there exists another category of pluripotent stem cells known as induced pluripotent stem cells (iPSCs), which are derived from somatic cells and can be reprogrammed to regain their pluripotent state (Fig. 1B) (Nishikawa et al. 2008). This groundbreaking technique of somatic cell reprogramming has facilitated the generation of various differentiated cell types with defined functions, such as cardiomyocytes, hematopoietic cells, islet cells, and neural cells by directly reprogramming somatic cells using lineage-specific master genes (Maherali and Hochedlinger 2008).

Moreover, cancer stem cells (CSCs) are a rare subpopulation of cells identified in tumors originating from diverse organs and tissues (Reya et al. 2001). These CSCs possess stem cell properties, such as self-renewal capacity and multipotency, and are essential for both tumor formation and maintenance (Fig. 1C). CSC also rely on functional local microenvironments for support, and reciprocal interactions are observed between CSC and their microenvironment (Lane et al. 2009).

TGFβ superfamily signaling are important for maintaining the self-renewal and differentiation of human stem cells (James et al. 2005; Pauklin and Vallier 2015; Mullen and Wrana 2017). TGFβ superfamily signaling also plays pivotal role in maintaining the pluripotency of iPSCs, and its modulation can effectively guide lineage-specific differentiation (Fei and Chen 2010; Wu et al. 2023). CSCs are regulated by TGFβ family signaling, which can be provided either through autocrine mechanisms or by factors present in their residing microenvironment (Nishimura et al. 2010; Scheel et al. 2011). While TGFβ family signaling governs the stemness and differentiation of normal and neoplastic stem cells, its effects exhibit diversity contingent upon cell types, microenvironmental factors, and physiological states (Watabe and Miyazono 2009). This review will primarily focus on elucidating the multifaceted roles of TGFβ superfamily signaling in governing various functions and properties of ESCs, somatic stem cells, iPSCs, and CSCs.

## TGFβ family signaling pathway

**Fig. 2:**
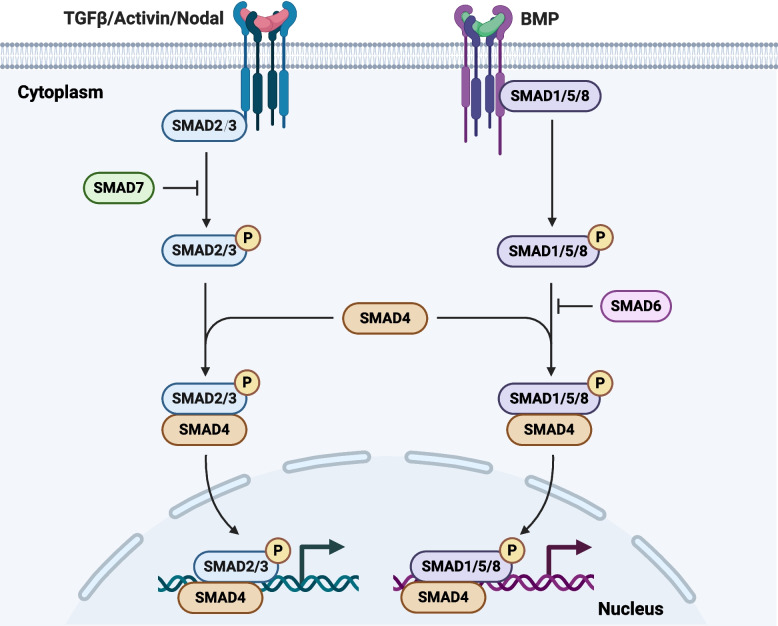
TGFβ family signaling pathway. TGFβ superfamily including TGFβ, Activin, Nodal, and BMP ligands, transmits signals through intrinsic type I and type II serine/threonine kinase receptors on the stem cell surface. Upon ligand stimulation, the type I receptor phosphorylates SMAD proteins (SMAD2 and SMAD3 for TGFβ/Activin/Nodal signaling; SMAD1, SMAD5, and SMAD8 for BMP signaling) and accumulate in the nucleus form complexes with a co-SMAD (SMAD4). Meanwhile, the signaling cascade induces inhibitory SMADs (SMAD6/SMAD7) to provide negative feedback on these pathways

The TGFβ family signaling play a crucial role in a wide range of biological processes (Massagué and Sheppard 2023). This superfamily consists of a diverse group of growth factors, including TGFβs, BMPs, Activins and Nodal (Fig. 2). These ligands bind to specific cell surface receptors, initiating a complex signaling cascade. There are two main types of receptors: type I and type II serine/threonine kinase receptors on cell surface (James et al. 2005; Fei and Chen 2010). When ligand binds to the receptor complex, it triggers a cascade of intracellular signaling events. The activated receptors phosphorylate and activate SMAD proteins, which are the main intracellular mediators of TGFβ superfamily signaling (Hata and Chen 2016). There are three classes of SMADs: receptor-regulated SMADs (SMAD2 and SMAD3 for TGFβ/Activin/Nodal signaling; SMAD1, SMAD5 and SMAD8 for BMP signaling), common mediator SMAD (SMAD4), and inhibitory SMADs (SMAD6/SMAD7) (Wrana 2000). R-SMADs are phosphorylated by the activated receptors and form complexes with Co-SMAD (SMAD4) (Wrana 2009). Inside the nucleus, SMAD complexes interact with other transcription factors and co-regulators to modulate the expression of genes that control stem cell fate (Massagué and Xi 2012; Beyer et al. 2013). Meanwhile, the signaling cascade induces inhibitory SMADs (SMAD6/SMAD7) provide negative feedback on these pathways (Nakao et al. 1997; Attisano and Wrana 2002; Avery et al. 2010).

## TGFβ family signaling in pluripotent stem cells

**Fig. 3:**
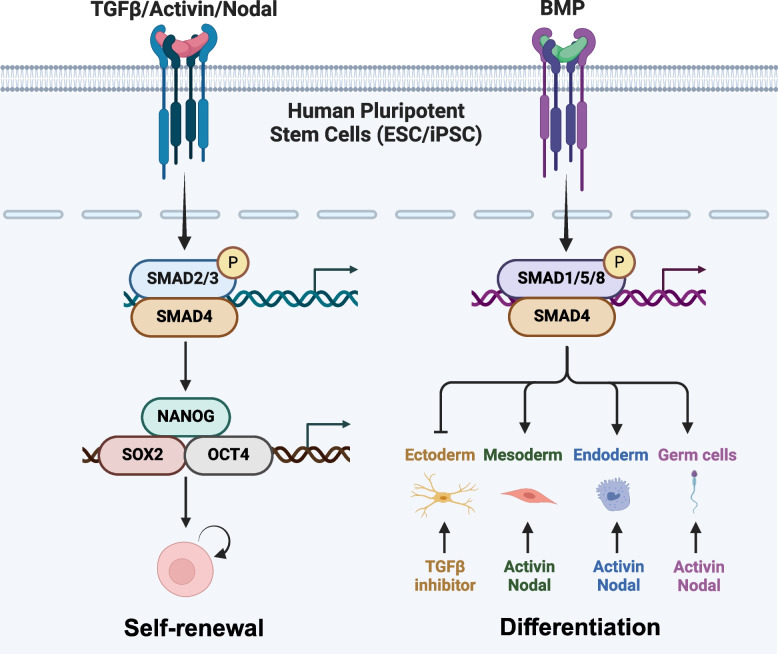
Role of TGFβ family signaling in human pluripotent stem cells. The signaling pathway mediated by TGFβ/Activin/Nodal occupies a pivotal role in the regulation of self-renewal in human embryonic stem cells and induced pluripotent stem cells. NANOG serves as a downstream target of the TGFβ/Activin/Nodal signaling cascade. Both TGFβ and BMP signaling inhibit ectoderm differentiation, while activating BMP/Activin/Nodal signaling mainly induces differentiation towards mesoderm, endoderm, and germ cells

TGFβ superfamily signaling regulates the self-renewal and differentiation of pluripotent stem cells including ESCs and iPSCs (Fig. 3) (Li et al. 2016b). In pluripotent stem cells, TGFβ signaling activates a set of transcriptional factors that are crucial for self-renewal and the prevention of premature differentiation. The pluripotency is maintained through the expression of transcription factors including *OCT4*, *NANOG* and *SOX2* (Boyer et al. 2005). TGFβ/Activin and fibroblast growth factor signaling pathways synergistically collaborate to suppress BMP signaling, sustain the expression of pluripotency-associated genes including *OCT4*, *NANOG* and *SOX2*, and facilitate long-term undifferentiated proliferation of ESCs (Xu et al. 2008; Greber et al. 2010). Both TGFβ and BMP-responsive SMADs exhibit binding affinity towards the *NANOG* proximal promoter. TGFβ signaling enhances *NANOG* promoter activity, while BMP signaling attenuates it (Suzuki et al. 2006; Xu et al. 2008; Galvin-Burgess et al. 2013). Nuclear accumulation of activated SMAD2 induced by TGFβ, Activin or Nodal was observed in undifferentiated ESCs, and exhibited a decline during early differentiation (James et al. 2005; Vallier et al. 2009; Sakaki-Yumoto et al. 2013a, b; Itoh et al. 2014). When ESCs initiate differentiation, the level of TGFβ/Activin/Nodal signaling decreases (Xiao et al. 2006; Xu et al. 2008). Inhibition of kinase activities of type I receptors for Activin-ALK4, TGFβ-ALK5, and Nodal-ALK7 using chemical kinase inhibitors such as SB431542 led to a reduction in the expression levels of pluripotency markers in human embryonic stem cells (Inman et al. 2002; James et al. 2005; Madhu et al. 2016). TGFβ signaling is essential for maintaining the pluripotency of human naïve pluripotent stem cells; however, inhibition of TGFβ signaling in naive human PSCs results in the downregulation of genes targeted by SMAD2/3 and the departure from pluripotency (Osnato et al. 2021). The generation of human ground state naive embryonic stem cells from embryos involves employing exogenous TGFβ and chemical inhibitors targeting ALK4/7, which induce a specific differentiation state in the resulting pluripotent stem cells (Gafni et al. 2013; Theunissen et al. 2014). Additionally, the downstream inhibition of DNA binding (ID) proteins, induced by BMP signaling, has been reported to maintain the self-renewal capacity of embryonic stem cells (Ying et al. 2003; Lasorella et al. 2014).

Pluripotent stem cells exhibit an extraordinary capacity to undergo differentiation into all three germ layers: ectoderm, mesoderm, and endoderm (Thomson et al. 1998). The initiation of the primitive streak (PS), which serves as the source of mesodermal and endodermal cells, constitutes the initial stage in ESC differentiation (Tam and Behringer 1997; Gadue et al. 2006). BMP4 can induce PS-like cells characterized by robust expression of the mesodermal marker Bra as well as posterior PS markers Msdp1 and Hoxb1 (Nostro et al. 2008). Multiple studies in the fields of development and stem cells have shown that Nodal/Activin signaling serves as the principal driver of definitive endoderm formation (Tiedemann et al. 2001; Kubo et al. 2004; D'Amour et al. 2005; Parashurama et al. 2007; Brown et al. 2011; Lee et al. 2015). Progression of the anterior PS population to definitive endoderm requires sustained activation of TGFβ/Nodal/Activin signals, consistent with the observation that Nodal/Activin signals are required for development of definitive endoderm in embryo (Gadue et al. 2006). Neural lineages are derived from ectoderm, BMP4 acts as a suppressor for neural differentiation induced in the PA6 stromal cells co-culture system or under serum-free culture conditions (Schulz et al. 2004). The induction of neural commitment is facilitated by inhibiting BMP signaling through Noggin or the small molecule BMP receptor kinase inhibitor dorsomorphin (Pera et al. 2004; Zhou et al. 2010). Simultaneous inhibition of both BMP and TGFβ/Activin/Nodal signaling using Noggin/SB431542 or dorsomorphin/SB431542 synergistically augmented the efficacy of neural differentiation (Chambers et al. 2009; Morizane et al. 2011). Human embryonic stem cells also exhibited the potential to differentiate into primordial germ cells upon stimulation with BMP. The addition of BMP4 to differentiating human embryoid bodies induced the expression of germ-cell specific marker genes, while the combined use of BMP7 and BMP8b showed additive effects with BMP4, resulting in a remarkable enhancement of germ cells (Kee et al. 2006; Panula et al. 2011). Moreover, Activin/Nodal showed promotion function in germ cell differentiation of human embryonic stem cells (Duggal et al. 2015; Jørgensen et al. 2018; Mishra et al. 2021).

## TGFβ family signaling in somatic stem cells

The somatic stem cells undergo symmetric or asymmetric cell divisions to generate both new stem cells and differentiated cell types, thereby replenishing dying cells and facilitating tissue regeneration (Miettinen et al. 1994; Shah and Khan 2021). The stemness of somatic stem cells is regulated by their intrinsic properties and external features that are maintained by their local cellular microenvironment (Spradling et al. 2001; Bendall et al. 2007; Ferraro et al. 2010, Voog and Jones 2010, Snippert and Clevers 2011). TGFβ family signaling pathways have been implicated in the maintenance and differentiation of various types of somatic stem cells (Watabe and Miyazono 2009; Sakaki-Yumoto et al. 2013a, b). This section focuses on elucidating the distinct roles of TGFβ family signaling in various stem cell populations, including hematopoietic stem cells, neural stem cells, hair follicle stem cells, intestinal stem cells and mesenchymal stem cells.

## TGFβ family signaling in hematopoietic stem cells

**Fig. 4:**
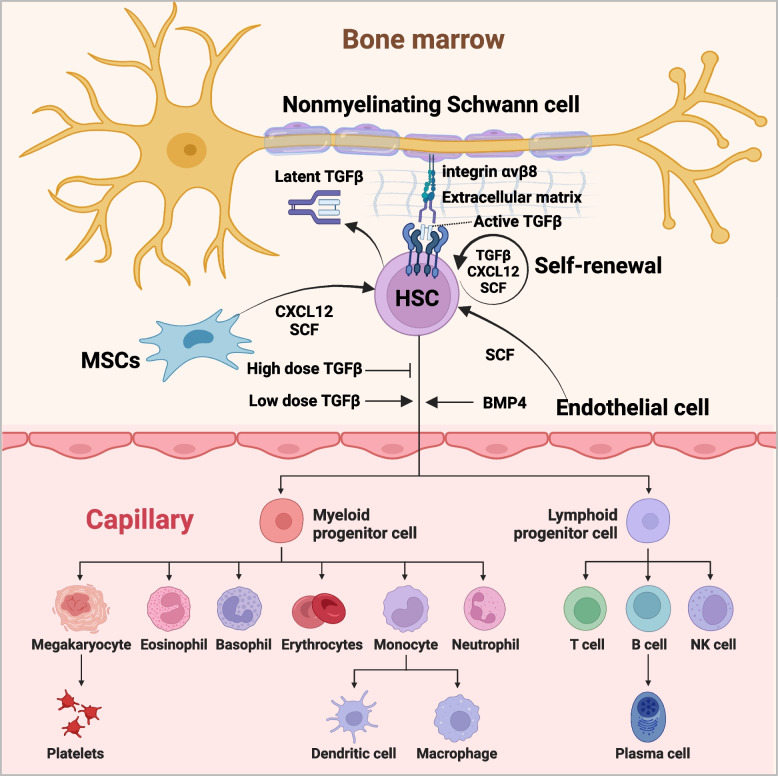
Role of TGFβ family signaling in hematopoietic stem cells. Hematopoietic stem cells (HSCs) primarily reside in the bone marrow and give rise to erythrocytes, thrombocytes, and leukocytes of both lymphoid and myeloid lineages. The maintenance of HSCs relies on TGFβ, CXCL12 and stem cell factor (SCF) provided by HSCs, mesenchymal stem cells (MSCs) and endothelial cells. The latent TGFβ produced by HSCs is activated by integrin αvβ8 in nonmyelinating Schwann cells, which binds to and cleaves the complex. BMP4 and low levels of TGFβ promote HSCs differentiation, while high levels of TGFβ inhibit it

Hematopoietic stem cells (HSCs) are primarily localized within the bone marrow and serve as the progenitors for erythrocytes, thrombocytes, and leukocytes encompassing both lymphoid and myeloid lineages (Dykstra et al. 2007; Wang and Wagers 2011). The maintenance of the HSC state is regulated by signals originating from the microenvironment, including mesenchymal stem cells, endothelial cells, osteoblasts, and sympathetic nerve fibers (Morrison and Scadden 2014). TGFβ family signaling in the hematopoietic system regulates the maintenance of quiescence and self-renewal capabilities in HSCs (Fig. 4) (Blank and Karlsson 2015). Endothelial cells are responsible for the production of stem cell factor (SCF), while mesenchymal stem cells (MSCs) produce both SCF and CXCL12, and HSCs generate TGFβ; all these factors are essential for HSC maintenance (Sugiyama et al. 2006; Yamazaki et al. 2009; Ding et al. 2012; Greenbaum et al. 2013). TGFβ serves as a potential signaling molecule in the bone marrow microenvironment to induce quiescence of hematopoietic stem cells (Yamazaki et al. 2009). Non-myelinating Schwann cells were found to contact a significant proportion of HSCs in the bone marrow and facilitate TGFβ activation through integrin αvβ8 expression, which directs metalloproteinases to cleave latent TGFβ (Fig. 4) (Yamazaki et al. 2011). TGFβ elicits highly variable biological responses and exerts both positive and negative effects on cellular proliferation, differentiation, and apoptosis (Larsson and Karlsson 2005; Ruscetti et al. 2005; Söderberg et al. 2009; Blank and Karlsson 2015). The response of HSCs to TGFβ stimulation demonstrates a biphasic pattern, whereby low concentrations of TGFβ induce stimulatory effects on HSC proliferation and differentiation, while high concentrations exert inhibitory effects (Kale and Vaidya 2004). BMP plays a crucial role in promoting the specification and expansion of HSCs during gastrulation in vertebrates, as well as being essential for their maintenance and proliferation in vitro (Bhatia et al. 1999; Kang et al. 2004). BMP9 has also been demonstrated as a potent synergistic factor in the generation and formation of hematopoietic progenitor cells (Ploemacher et al. 1999). BMP4, in conjunction with cytokines, facilitate hematopoietic specification, differentiation, and proliferation of human embryonic stem cells (Park et al. 2004).

## TGFβ family signaling in neural stem cells

**Fig. 5:**
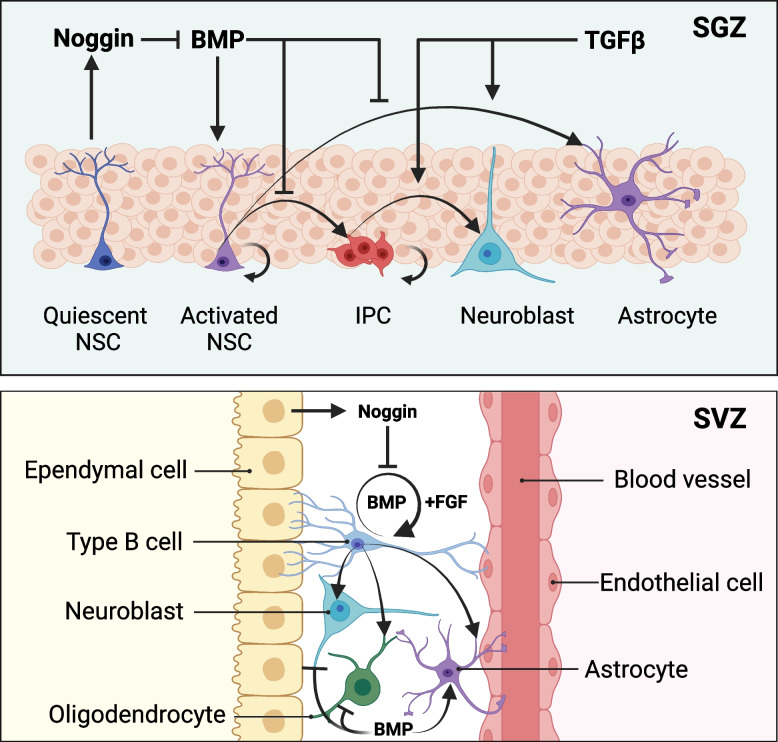
Role of TGFβ family signaling in neural stem cells. BMP signaling activates NSCs in the subgranular zone (SGZ), while Noggin produced by quiescent NSCs inhibits BMP signaling. Inhibition of BMP signaling leads to the formation of intermediate progenitor cells (IPCs), which differentiate into neuroblasts and astrocytes in vivo. The NSCs in the subventricular zone (SVZ), known as type B cells, are maintained as slowly cycling NSCs through BMP and fibroblast growth factor (FGF) signaling. The production of Noggin by ependymal cells hinders the maintenance of NSC state regulated by BMP signaling. Upon initiation of differentiation, BMP signaling pathway facilitates astrocyte differentiation while repressing neural and oligodendrocyte lineages. Later in development, TGFβ facilitates the differentiation and lineage expansion of established progenitors

The adult brain contains two populations of neural stem cells (NSCs) located in the subgranular zone (SGZ) of the hippocampal dentate gyrus and the subventricular zone (SVZ) lining the lateral ventricles (Alvarez-Buylla et al. 2002; Kriegstein and Alvarez-Buylla 2009). The NSCs have the ability to differentiate into intermediate progenitor cells (IPCs), also known as transit-amplifying or neural progenitor cells, which subsequently generate neurons, astrocytes, and oligodendrocytes (Reynolds and Weiss 1992). NSCs can be cultured in vitro as neurospheres and differentiated into both neuronal and glial cells; however, it has been suggested that this differentiation behavior is influenced by the specific culture conditions employed (Marshall, Laywell et al. 2006). TGFβ signaling pathway plays pivotal roles in the maintenance and proliferation of neural stem cells (Falk et al. 2008; Seuntjens et al. 2009). While the effects of TGFβ family signaling in NSCs are highly context-dependent and can vary depending on the developmental stage and specific signaling components involved. The canonical TGFβ pathway in midbrain development acts as a suppressor of Wnt-induced proliferation and expansion of neuroepithelial cells, which are the NSCs involved in early brain development (Fig. 5) (Falk et al. 2008). ALK5-dependent TGFβ signaling plays a crucial role in regulating the later stages of adult hippocampal neurogenesis, facilitating stem cell quiescence and promoting neurogenesis within the adult neurogenic niche (He et al. 2014; Kandasamy et al. 2014). Considering the heightened levels of TGFβ1 observed in the context of aging and neurodegenerative disorders, targeting TGFβ1 signaling emerges as a promising molecular strategy for future interventions in such pathological conditions (Kandasamy et al. 2014).

BMPs induce various biological responses in embryonic neural stem cells, with BMP2/4 playing a crucial role in promoting neuroepithelial proliferation during early stages of embryonic central nervous system development (Panchision et al. 2001). During later stages of development, BMPs stimulate the differentiation of neural stem cells into neurons and astrocytes (Gross et al. 1996). BMP signaling pathway plays a crucial role in regulating the multipotency of neural stem cells in both SGZ and SVZ; however, divergent mechanisms have emerged regarding its utilization by these two distinct stem cell populations (Alvarez-Buylla and Lim 2004; Chen and Panchision 2007). The NSCs in the SVZ, known as B cells, express BMP2/4/7 and are located in the ventricular walls. The Noggin secreted by the adjacent ependymal cells acts as an inhibitory factor for BMP in B cells (Lim et al. 2000; Bonaguidi et al. 2008). The BMP signaling pathway helps maintain the B-cell state, but once differentiation begins, it promotes astrocyte differentiation while suppressing neural and oligodendrocyte fates (Lim et al. 2000). The downstream targets of BMP signaling, ID1/3, are essential for the synchronization of stemness and anchorage to the neural stem cell niche (Niola et al. 2012).

## TGFβ family signaling in hair follicle stem cells

**Fig. 6:**
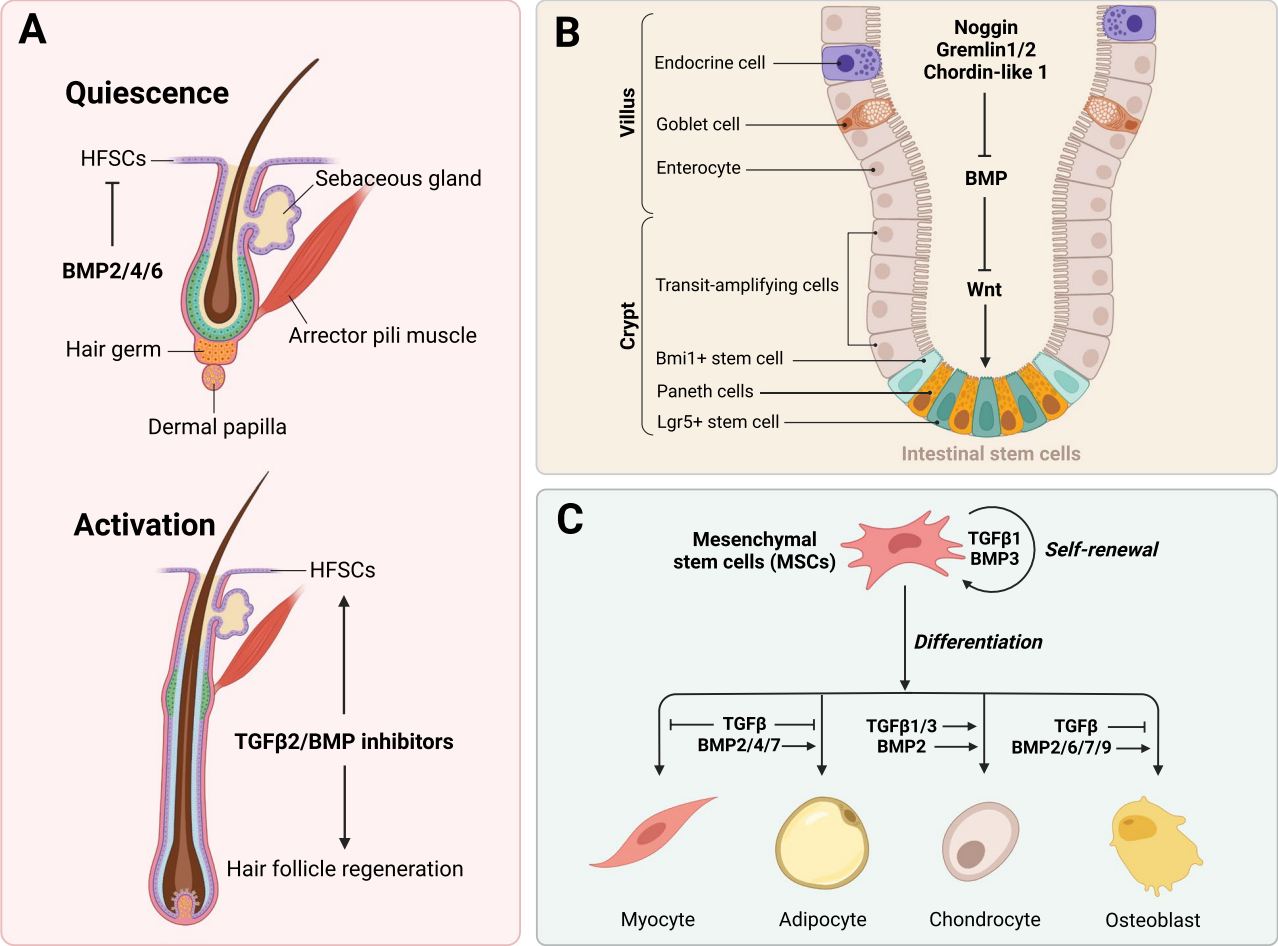
Role of TGFβ family signaling in hair follicle, intestinal and mesenchymal stem cells. **A** BMP2/4/6 effectively inhibits hair follicle stem cell (HFSC) proliferation and maintains them in a quiescent state. The expression of TGFβ2 and Noggin by dermal papilla cells inhibit BMP signaling and promotes HFSC proliferation in an activation state.** B** Wnt signaling promotes intestinal stem cells (ISCs) proliferation in the crypts, while BMP signaling inhibits stem cells proliferation. The inhibitors of BMP signaling, such as Noggin, Gremlin 1/2, and Chordin-like 1, promote the maintenance of ISCs. **C** TGFβ1 and BMP3 promotes mesenchymal stem cells (MSCs) maintenance, while other BMPs promote adipocyte and osteocyte differentiation. TGFβ1/3 and BMP2 work together to promote chondrocyte differentiation, whereas TGFβ inhibit myoblast differentiation

Throughout adulthood, hair follicle stem cells (HFSCs) undergo dynamic and synchronized cycles of telogen, anagen and catagen (Millar 2002; Schmidt-Ullrich and Paus 2005). In the telogen phase, HFSCs are quiescent and reside in a specialized microenvironment called the hair bulge, which is directly adjacent to the underlying dermal papilla (DP), a signaling center for HFSCs (Cotsarelis et al. 1990). The follicle is maintained in telogen primarily by BMP secretion from surrounding cell populations (Fig. 6A). Adipocytes express BMP2, dermal fibroblasts express BMP4, and keratin 6 + inner bulge cells express BMP6, suppressing HFSCs proliferation (Plikus et al. 2008; Hsu et al. 2011; Oshimori and Fuchs 2012). The transition from telogen to anagen is characterized by the suppression of BMP signaling, leading to the proliferation and differentiation of HFSCs. The inactivation of the *BMPR1A* gene in postnatal skin epithelium triggers the activation and proliferation of quiescent HFSCs, resulting in the depletion of slowly proliferating cells (Zhang et al. 2006; Kobielak et al. 2007). Consistent with this observation, BMP inhibitory factors secreted by neighboring niche cells have been implicated in the initiation of anagen (Kulessa et al. 2000; Botchkarev et al. 2001). TGFβ2 isoform plays a distinct role, unique among other TGFβ isoforms, in the induction of hair follicle morphogenesis and is both essential and adequate for facilitating this process (Foitzik et al. 1999). Additionally, TGFβ2 antagonizes BMP signaling in HFSCs by upregulating the expression of TMEFF1, a potent antagonist of the BMP pathway. This mechanism effectively restricts and attenuates the responsiveness of HFSCs to BMP signals within their niche (Oshimori and Fuchs 2012). Mesenchymal DP cells transmit paracrine TGFβ2 to epidermal HFSCs prior to their activation, thereby initiating TGFβ/SMAD2/3 signaling cascade that stimulates HFSCs and promotes tissue regeneration (Oshimori and Fuchs 2012). TGFβ1 plays a crucial role in regulating catagen induction in vivo by inhibiting keratinocyte proliferation and promoting apoptosis, suggesting it as a potential target for treating hair growth disorders caused by premature or delayed catagen development (Foitzik et al. 2000; Soma et al. 2003; Mesa et al. 2015).

## TGFβ family signaling in intestinal stem cells

The intestinal stem cells (ISCs) are located within the crypts of both the small intestine and colon, providing a continuous supply of intestinal epithelial cells that subsequently migrate to the villi (Fig. 6B) (Leblond and Stevens 1948; Creamer et al. 1961; Flier and Clevers 2009). Intestinal crypts contain two types of stem cells: Lgr5 + stem cells, which proliferate rapidly and produce intestinal epithelial cells, and Bmi1 + stem cells, which are quiescent and play a crucial role in regenerating the intestinal epithelium after injury (Barker et al. 2007; Sangiorgi and Capecchi 2008). The proliferation and differentiation of ISCs are regulated by factors secreted from the underlying mesenchymal layer, including fibroblasts, enteric neurons, blood vessels, and extracellular matrix components (Takeda et al. 2011). Wnt signaling promotes the proliferation of stem cells and transiently amplifying cells in the crypts, while BMP signaling functions as a negative regulator (Gregorieff and Clevers 2005; Gregorieff et al. 2005). The intravillus mesenchyme shows high expression of BMP4, and phosphorylation and nuclear localization of SMADs have been observed in differentiated villus epithelial cells and ISCs (Haramis et al. 2004; He et al. 2004).The BMP antagonists Noggin, Gremlin1 and 2, and Chordin-like 1 exhibit elevated expression levels in the crypts or their underlying mesenchyme (He et al. 2004; Kosinski et al. 2007). The loss of BMP signaling in the intestinal epithelium leads to an expansion of crypts, indicating that BMP signaling suppresses crypt formation and inhibits the expansion of the stem-cell niche (Haramis et al. 2004; He et al. 2004; Davis et al. 2015). BMP signaling is crucial for maintaining the quiescence of Bmi1 + stem cells and promoting terminal differentiation of intestinal epithelial villi, potentially by antagonizing Wnt signaling, which stimulates the proliferation of the Lgr5 + stem cell population (He et al. 2004).

## TGFβ family signaling in mesenchymal stem cells

The mesenchymal stem cells (MSCs) can be achieved from various adult tissues, encompassing connective tissue, adipose tissue, muscle, bone marrow, blood, placenta and umbilical cord (Pittenger et al. 1999; Shi et al. 2005; Phinney and Prockop 2007). MSCs are of clinical significance due to their easy accessibility from adult patients, remarkable capacity for self-renewal, multipotency, and potent immunomodulatory effects on immune cells (Caplan 2000; Chen et al. 2022).The differentiation potential of MSCs is influenced by the specific tissue environment from which they are derived, and it is determined by their localization and microenvironment in vivo (Fig. 6C) (Roelen and Dijke 2003). Bone marrow-derived MSCs possess the capacity to undergo differentiation into osteoblasts, adipocytes, chondrocytes, myocytes, and stromal cells that support hematopoiesis (Pittenger et al. 1999). Muscle-derived MSCs have the capacity to differentiate into myogenic, osteogenic, chondrogenic, and adipogenic lineages (Williams et al. 1999). MSCs derived from adipose tissue possess the capacity to undergo differentiation into osteocytes, chondrocytes, adipocytes, and myocytes (Zuk et al. 2002).The proliferation of human MSCs is stimulated by Wnt or TGFβ signaling pathways (Boland et al. 2004; Jian et al. 2006). Transcriptomic analysis suggests that Wnt signaling plays a crucial role in maintaining the self-renewal and proliferation of primitive MSCs, whereas TGFβ signaling is implicated in the gradual decline of self-renewal capacity and initiation of senescence (Mazzella et al. 2023). TGFβ1 induces SMAD3-dependent nuclear accumulation of β-catenin in MSCs, promoting their proliferation. Conversely, BMP2 inhibits WNT3A signaling and MSC proliferation by interacting with Dishevelled-1, a component of the Wnt pathway (Liu et al. 2006). BMP3 signaling enhances MSC proliferation through the TGFβ/Activin pathway by inducing SMAD2 phosphorylation (Stewart et al. 2010). TGFβ signaling has also been implicated in orchestrating the differentiation fate of MSCs, promoting chondroblast differentiation during early stages while subsequently inhibiting osteoblast maturation (Roelen and Dijke 2003; Grafe et al. 2018). Pharmacological inhibition of TGFβ signaling significantly enhances the maturation process of osteoblasts (Maeda et al. 2004). The presence of BMPs has been demonstrated to facilitate the differentiation of MSCs into osteocytes, while also promoting the development of adipocytes and chondrocytes (Ahrens et al. 1993; Luu et al. 2007). The signaling of BMP2/4/7 promotes adipocyte differentiation by inducing the expression of PPARγ, whereas TGFβ and Activin A exert inhibitory effects on adipocyte development (Choy et al. 2000; Lee et al. 2000; Jin et al. 2006). The adipocytes can be divided into two categories: white adipocytes synthesize white fat to store triglycerides, while brown adipocytes promote energy expenditure and develop brown fat (Cristancho and Lazar 2011). BMP4 is widely acknowledged as a protein that facilitates the differentiation of white adipocytes, whereas BMP7 has emerged as a specific regulator of brown adipogenesis, suggesting that targeting BMP7 could represent a potential therapeutic approach for obesity (Tseng et al. 2008; Zamani and Brown 2011). TGFβ is also known for its role in driving the differentiation of MSCs into myofibroblasts, which contribute to excessive collagen deposition and fibrosis (Popova et al. 2010). Manipulating TGFβ family signaling pathways can enhance MSC functionality for tissue repair and tissue remodeling, with significant implications for MSC-based therapies and regenerative medicine.

## TGFβ family signaling in human iPSC reprogramming

Reprogramming of human somatic cells into iPSCs can be achieved by overexpressing specific transcription factors including OCT4, SOX2, KLF4, and CMYC (Takahashi et al. 2007; Li et al. 2016a, b; Guo et al. 2019). The molecular profiling identified three distinct transcriptional phases during reprogramming: essential early phase of mesenchymal-to-epithelial transition (MET) initiation, intermediate maturation phase acquires pluripotent competency, and stabilized pluripotent state with full pluripotency network (Samavarchi-Tehrani et al. 2010; Mullen and Wrana 2017).

A large number of experimental studies have proven that the TGFβ signaling pathway is a key signaling pathway in iPSC reprogramming (Li et al. 2016a, b; Fan et al. 2024). TGFβ facilitates the induction of epithelial-to-mesenchymal transition (EMT) in cells, exerting inhibitory effects on iPSC reprogramming (Lin et al. 2009; Li et al. 2010). Inhibiting TGFβ/Activin/Nodal type I receptor kinases enhances iPSC induction and eliminates the need for introducing SOX2 expression (Ichida et al. 2009). Additionally, treatment of partially reprogrammed iPSCs with TGFβ receptor inhibitor significantly induces NANOG expression, promoting full reprogramming (Ichida et al. 2009; Maherali and Hochedlinger 2009). Reprogramming efficiency of iPSCs can be attenuated by treatment with TGFβ or introduction of an activated type I TGFβ receptor, whereas enhancement of iPSCs reprogramming processes can be achieved through inhibition of TGFβ type II receptor expression by miRNAs (Li et al. 2011; Miyoshi et al. 2011; Subramanyam et al. 2011). BMP signaling promotes iPSCs reprogramming by inducing MET and antagonizing TGFβ stimulation in specific contexts (Li et al. 2010; Samavarchi-Tehrani et al. 2010).

TGFβ signaling is also essential in maintaining the self-renewal and pluripotency of human iPSCs similar like ESCs (Fig. 3), and its modulation can direct lineage-specific differentiation (Robinton and Daley 2012; Moradi et al. 2019). The optogenetic modulation of TGFβ signaling can precisely control the differentiation of human iPSCs into the mesenchymal lineage (Wu et al. 2023).The synergistic action of two inhibitors of SMAD signaling (Noggin and SB431542) is sufficient to induce iPSCs differentiate to neural tissue and are patternable to dopaminergic neurons and motoneurons (Chambers et al. 2009). Inhibition of TGFβ signaling pathway promotes differentiation of human iPSC-derived brain microvascular endothelial-like cells (Yamashita et al. 2020). A8301, a TGFβ signaling inhibitor, is sufficient to switch the cell fate from iPSCs into neural progenitor cells in OCT4/SOX2/KLF4/MYC-mediated human urine cells reprogramming (Wang et al. 2016a, b). BMPs are commonly combined with other growth factors (e.g., FGF, Wnt, or VEGF) and/or pathway inhibitors like small molecule inhibitors targeting TGFβ or Wnt signaling (SB431542 and CHIR99021), to induce iPSC differentiation towards specific cell types (Sánchez-Duffhues and Hiepen 2023). By manipulating these pathways, researchers can control iPSC behavior and enhance their utility in regenerative medicine and therapeutic applications.

## TGFβ family signaling in cancer stem cells

Cancer stem cells (CSCs) represent a sub-population of cells within a tumor that have the ability to self-renew and give rise to the heterogeneous cell types found in the tumor (Ayob and Ramasamy 2018). CSCs play a pivotal role in tumorigenesis, progression, metastasis, relapse, and resistance mechanisms, exerting a profound impact on the aggressiveness of cancer (Reya et al. 2001; Graham et al. 2002; Bao et al. 2006; Nunes et al. 2018; Huang et al. 2020). TGFβ signaling can have a dual role in relation to CSCs. Initially, TGFβ can act as a tumor suppressor (Gu and Feng 2018). In normal and pre-malignant stem cells, it can maintain genomic stability and inhibit excessive self- renewal (Futakuchi et al. 2019). For example, TGFβ signaling initially restricts their proliferation in intestinal stem cells, but as tumors progress, cancer cells can hijack the TGFβ signaling pathway, where mutations can lead to constitutive activation of downstream pathways, and this abnormal activation promotes the self-renewal of CSCs, enabling them to continuously generate more cancer cells and contribute to tumor growth, metastasis, and drug resistance (Chen et al. 2024).

EMT process leads to an augmentation in the population of cells exhibiting stem cell properties, commonly referred to as CSCs (Dongre and Weinberg 2019, Celià-Terrassa and Jolly 2020). During cancer progression, TGFβ signaling pathway plays an important role in orchestrating EMT, facilitating the generation of stem cells, development of resistance to anti-cancer drugs, induction of genomic instability, and establishment of localized immunosuppression (Katsuno et al. 2013; Moses et al. 2016; Katsuno and Derynck 2021). TGFβ is a major driver in promoting the differentiation of mesenchymal stem cells derived from bone marrow and adipose tissue into cancer-associated fibroblasts (Jotzu et al. 2010; Yang et al. 2012). TGFβ can also induce the differentiation of mesenchymal stem cells into endothelial cells, promoting a pro-angiogenic microenvironment and facilitating tumor angiogenesis (Li et al. 2016; Batlle et al. 2019). In breast cancer, TGFβ-induced activation of the SMAD pathway can up-regulate genes that enhance the self-renewal capacity of CSCs (Wang et al. 2023). TGFβ superfamily signaling pathway regulates the state of pluripotency in human stem cells and their capacity to generate well-structured telencephalic organoids, thereby enhancing the formation of sophisticated brain disease models (Watanabe et al. 2022).

In cancer therapy, hematopoietic stem cell gene therapy targeting TGFβ augmented the efficacy of irradiation therapy in a preclinical glioblastoma model (Andreou et al. 2021). Inhibition of TGFβ enhances the functionality of the hematopoietic stem cell niche and mitigates cancer-related anemia (Wang et al. 2021). In a preclinical glioblastoma model, researchers demonstrated that targeting the αv integrin/TGFβ axis enhances natural killer cell functionality against glioblastoma stem cells (Shaim et al. 2021). Inhibitory effect of TGFβ1-blocking peptides P17 and P144 on EMT in CSCs was demonstrated, leading to the suppression of liver metastasis in a preclinical model of advanced colorectal cancer (Zubeldia et al. 2013). In Table 1, we summarize the studies that demonstrate the significant involvement of TGFβ superfamily signaling in the maintenance and differentiation of various cancer stem cells, offering promising clinical applications for targeting TGFβ family signaling to effectively impede cancer progression (Aigner and Bogdahn 2008; Watabe and Miyazono 2009; Caja et al. 2012; Sakaki-Yumoto et al. 2013a, b; Caja et al. 2015).

**Table 1:** Roles of TGFβ family signaling pathway in various cancer stem cells

**Cancer**	**Function**
**Glioma**	TGFβ increases glioma-initiating cell self-renewal through the induction of LIF in human glioblastoma (Peñuelas et al. 2009)
	TGFβ promotes Sox4 and SOX2 expression and maintains stemness of glioma-initiating cells (Ikushima et al. 2009)
	BMP4 suppresses glioblastoma stem cell proliferation and promotes differentiation (Piccirillo et al. 2006)
	TβR2 serves as a novel regulator governing the properties of glioblastoma stem cells (Narushima et al. 2016)
	BMP signaling orchestrates the quiescence of glioma stem cells and confers resistance to radiation and temozolomide chemotherapy in glioblastoma (Sachdeva et al. 2019)
	NOX4 regulates TGFβ signaling to promote the proliferation and self-renewal of glioblastoma stem cells (García-Gómez et al. 2022)
	BMPRIB/ALK6 promotes terminal differentiation of brain cancer stem cells (Lee et al. 2008)
**Leukemia**	TGFβ controls Foxo3a localization and maintains stem cell-like properties of leukemia initiating cells (Naka et al. 2010)
	SMAD4 protects Hoxa9-induced transformation of normal hematopoietic stem cells and inhibits leukemia initiation (Quéré et al. 2011)
**Breast cancer**	TGFβ reduces the size of the putative cancer stem cell population and inhibits tumor formation (Tang et al. 2007)
	BMP2/7 suppresses breast cancer stem cell subpopulation and bone metastases formation (Buijs et al. 2012)
	TGFβ regulates the ILEI/LIFR signaling axis and promote self-renewal of breast cancer stem cells (Woosley et al. 2019)
	Treatment with TGFβ1 at a low concentration for a short time may induce the auto-induction of TGF β1 in breast cancer stem cells (Hariyanto et al. 2021)
**Diffuse-type gastric cancer**	TGFβ inhibits ATP binding cassette subfamily G member 2 (ABCG2) transcription and decreases the cancer-initiating cell populations within the cancer (Ehata et al. 2011)
	TGFβ reduces the expression of ALDH1 and REG4 to suppress cancer-initiating cell populations and tumorigenicity (Katsuno et al. 2012)
**Pancreatic cancer**	Nodal/Activin signaling promotes self-renewal and tumorigenicity in pancreatic cancer stem cells (Lonardo et al. 2011)
	Bromodomain containing 9 controls the TGFβ/Activin/Nodal pathway to regulate self-renewal, differentiation of human embryonic stem cells and cancer cell progression (Wang et al. 2023)
**Colorectal cancer**	BMP4 promotes colorectal cancer stem cell differentiation and enhances their chemo-sensitization (Lombardo et al. 2011)
	The downstream targets ID1/3 of BMP signaling regulate the self-renewal capacity of human colon cancer-initiating cells (O'Brien et al. 2012)
	Activin promotes self-renewal of colorectal cancer stem cells and facilitates tumor progression (Liu et al. 2016)
	BMP2 enhances radiosensitivity in colorectal cancer stem cells (Mahmoudi et al. 2024)
**Ovarian cancer**	TGFβ-induced transglutaminase 2 promotes ovarian tumor metastasis by inducing EMT and a cancer stem cell phenotype (Cao et al. 2012)
	TGFβ-mediated LEFTY/Akt/GSK-3β/Snail axis modulates EMT and cancer stem cell properties in ovarian clear cell carcinomas (Matsumoto et al. 2018)
**Prostate cancer**	BMP7 inhibits prostate cancer stem-like cell growth by activating p38 and increasing p21/NDRG1 expression (Kobayashi et al. 2011)
**Squamous cell carcinomas**	TGFβ signaling pathway limits the self-renewal and proliferation of cancer stem cells (Schober and Fuchs 2011)
**Esophageal cancer**	TGFβ1 inhibitors can reduce the migration and invasion abilities of esophageal cancer stem cells (Yue et al. 2015)
**Liver cancer**	TGFβ1 regulates liver cancer cells by modulating the expression of liver cancer stem cell markers such as CD133 and EpCAM (Wang et al. 2016a, b)

## TGFβ family signaling in stem cell therapy

Stem cell therapy, represents a regenerative medicine approach employed for the investigation and treatment of various human diseases (De Luca et al. 2019). Recent progress in stem cell technology has opened up a new avenue for patients afflicted with diseases and disorders that remain untreated (Hoang et al. 2022). TGFβ family signaling have been investigated in the context of stem cell therapy for modulating various diseases and offering promising therapeutic approaches. Targeting these pathways offers potential to enhance therapeutic outcomes by directing stem cell behavior more precisely.

In the context of neurodegenerative diseases such as Alzheimer's disease and Parkinson's disease, TGFβ signaling in NSCs is disrupted (Krieglstein 2013) (von Bernhardi et al. 2015; Karampetsou et al. 2022). Normally, TGFβ is involved in the self-renewal and maintenance of NSCs, but in the diseased brain, altered TGFβ levels are evident as in Alzheimer's disease where amyloid-beta plaques and tau tangles interfere with TGFβ signaling in NSCs, and reduced TGFβ signaling may lead to a decrease in the self-renewal capacity of NSCs, impairing the brain's ability to regenerate neurons and repair damaged neural tissue and contributing to the progressive loss of cognitive function (Meyers and Kessler 2017). Photoactivation of the TGFβ/SMAD signaling pathway improves neurogenesis in neural stem cells within an Alzheimer's disease model (Wu et al. 2021).

In fibrotic diseases such as liver fibrosis and pulmonary fibrosis, TGFβ signaling plays a central role in the behavior of mesenchymal stem cells (MSCs) (Taherian et al. 2024). In fibrotic conditions, MSCs are recruited to the injury site where TGFβ from damaged and inflammatory cells activates them and induces differentiation into myofibroblasts, whose enhanced self-renewal due to TGFβ signaling leads to excessive collagen production that disrupts normal tissue architecture and function (Qin et al. 2023). In liver fibrosis, the abnormal TGFβ-mediated self-renewal of activated stellate cells (a type of MSC-like cell) results in the deposition of fibrotic tissue and the progressive loss of liver function (Dewidar et al. 2019).

In type I diabetes, abnormal TGFβ signaling disrupts the cues for islet stem cell differentiation, high levels of TGFβ inhibit the differentiation of islet stem cells into functional β-cells by modulating crucial transcription factors and signaling molecules, impairing the body's ability to regenerate insulin-producing cells and contributing to the persistence of hyperglycemia (Lee et al. 2021). By manipulating TGFβ signaling pathways, researchers may be able to enhance the efficacy of stem cell therapies and promote the differentiation of stem cells into β-cells to improve insulin production and manage hyperglycemia (Brown and Schneyer 2021).

In the field of stem cell-based therapies, the immunomodulatory properties of TGFβ are of great importance (Akhurst and Hata 2012). MSCs secrete TGFβ which can act on immune cells like T-lymphocytes, B-lymphocytes, and NK cells to down-regulate their activation and proliferation and inhibit the production of pro-inflammatory cytokines such as IL-2 and IFN-γ by T-cells, thereby reducing the inflammatory environment (Atiya et al. 2020). Disruption of the TGFβ signaling is necessary for effective killing of hepatocellular carcinoma by human iPSC-derived NK cells (Thangaraj et al. 2024). Combined blockade of TGFβ and PD-L1 facilitates the expansion and differentiation of CD8 T cells with stem cell-like properties in immune-excluded tumors (Castiglioni et al. 2023).

## TGFβ family signaling in tissue regeneration

Regenerative medicine is a novel and promising mode of therapy for patients who have limited or no alternative treatment options for their illness (Mousaei Ghasroldasht et al. 2022). TGFβ superfamilies are important mediators of tissue repair and regeneration as TGFβ secreted by stem cells or other cells in the damaged tissue microenvironment during tissue repair processes helps in recruiting other cells to the injury site (Xu et al. 2018). Researchers showed that MSCs therapy decreased fibrosis in a murine full thickness wound healing model, which correlated with a reduced TGFβ1/TGFβ3 ratio from wound lysates (Qi et al. 2014). TGFβ3 has been reported promote scarless healing in the fetus and reduced scarring in adults, which offer a scar-reducing therapy for acute and chronic wounds and fibrosing disorders (Lichtman et al. 2016). Periodontal ligament stem cells and gingival mesenchymal stem cells encapsulated in TGFβ3-loaded Arg-Gly-Asp modified alginate microspheres are promising candidates for tendon tissue regeneration (Moshaverinia et al. 2014). Exogenous addition of TGFβ protein in vivo during muscle regeneration results in a loss of muscle function while inhibition of TGFβR2 induces the formation of giant myofibers (Girardi et al. 2021). BMP7 has been reported significantly enhanced the odontoblastic differentiation and mineralized nodule formation in dental pulp stem cells, which are crucial for dental pulp regeneration (Suzuki et al. 2011; Liang et al. 2021). Inhibition of TGFβ signaling via suppressing muscle segment homeobox2 enhances hematopoietic differentiation of human ESCs, providing valuable insights into the efficient generation of functional blood cells from pluripotent stem cells for regenerative medicine (Wang et al. 2020).

## Conclusions and perspectives

Recent advancements in stem cell research have paved the way for promising therapeutic approaches to address a wide range of diseases, including diverse types of cancer as well as metabolic and neurodegenerative disorders. The development of human induced pluripotent stem cells offers transformative potential for medical practice, promising to revolutionize therapeutic approaches. To effectively harness the therapeutic potential of stem cells, a comprehensive understanding of both extrinsic and intrinsic factors governing self-renewal and differentiation processes is imperative. Previous studies have demonstrated the crucial roles of the TGFβ family in governing stem cell self-renewal and differentiation across various developmental stages and diseases. One of the major challenges in using TGFβ family signaling in stem cell therapy is achieving precise control of the signaling pathways. Since these pathways are complex and involved in multiple cellular processes, over-or under-activation can lead to unwanted outcomes.

TGFβ family signaling often crosstalk with other signaling pathways in stem cells, such as Wnt, Notch, and Hedgehog pathways. Future research needs to focus on deciphering these complex crosstalk mechanisms to better design stem cell-based therapies. The investigation of dysregulated regulation in TGFβ family signaling pathways and the crosstalk between other signaling pathways governing the proliferation and differentiation of cancer stem cells holds significant interest for future research to identify suitable targets for personalized cancer therapies. Insights gained from these investigations are anticipated to facilitate the development of innovative therapeutic and prognostic modalities for addressing a range of cancers.

Although signaling within the TGFβ family has demonstrated substantial potential in pre-clinical studies related to stem cell therapy and tissue regeneration, there remain significant obstacles in clinical translation. The delivery of TGFβ family factors or modulators to the target tissue in a safe and effective manner also needs to be addressed. This could include the use of nanoparticles, gene therapy vectors, or engineered stem cells that can secrete these factors in a controlled manner at the target site. Additionally, long-term safety and efficacy evaluations in human patients are required to ensure the successful application of TGFβ family signaling in stem cell-based therapy and tissue regeneration. In conclusion, the elucidation of the complex regulatory networks involving TGFβ family signaling in stem cells is of utmost importance for future study to uncover the full therapeutic potential of stem cells in both regenerative medicine and cancer therapy.

## Data Availability

Not applicable.

## Abbreviations

ABCG2: ATP Binding Cassette Subfamily G Member 2
ALDH: Aldehyde dehydrogenases
BMP: Bone morphogenetic protein
CSC: Cancer stem cell
DP: Dermal papilla
EMT: Epithelial-mesenchymal transition
ESC: Embryonic stem cell
FGF: Fibroblast growth factor
HFSC: Hair follicle stem cell
HSC: Hematopoietic stem cell
ICM: Inner cell mass
ID: Inhibition of DNA binding protein
ILEI: Interleukin-like EMT inducer
IPC: Intermediate progenitor cell
iPSC: Induced pluripotent stem cell
ISC: Intestinal stem cell
LIF: Leukemia inhibitory factor
MSC: Mesenchymal stem cell
MET: Mesenchymal-to-epithelial transition
NDRG1: N-Myc Downstream Regulated 1
NOX4: NADPH Oxidase 4
NSC: Neural stem cell
PS: Primitive streak
REG4: Regenerating Family Member 4
SCF: Stem-cell factor
SGZ: Subgranular zone
Sox: SRY-box transcription factor
SVZ: Subventricular zone
TGFβ: Transforming growth factor β

## References

[CR1] Ahrens M, Ankenbauer T, Schröder D, Hollnagel A, Mayer H, Gross G. Expression of human bone morphogenetic proteins-2 or -4 in murine mesenchymal progenitor C3H10T1/2 cells induces differentiation into distinct mesenchymal cell lineages. DNA Cell Biol. 1993;12(10):871–80. 10.1089/dna.1993.12.871. 8274220 10.1089/dna.1993.12.871

[CR2] Aigner L, Bogdahn U. TGF-beta in neural stem cells and in tumors of the central nervous system. Cell Tissue Res. 2008;331(1):225–41. 10.1007/s00441-007-0466-7. 17710437 10.1007/s00441-007-0466-7

[CR3] Akhurst RJ, Hata A. Targeting the TGFβ signalling pathway in disease. Nat Rev Drug Discov. 2012;11(10):790–811. 10.1038/nrd3810. 23000686 10.1038/nrd3810 PMC3520610

[CR4] Alvarez-Buylla A, Lim DA. For the long run: maintaining germinal niches in the adult brain. Neuron. 2004;41(5):683–6. 10.1016/s0896-6273(04)00111-4. 15003168 10.1016/s0896-6273(04)00111-4

[CR5] Alvarez-Buylla A, Seri B, Doetsch F. Identification of neural stem cells in the adult vertebrate brain. Brain Res Bull. 2002;57(6):751–8. 10.1016/s0361-9230(01)00770-5. 12031271 10.1016/s0361-9230(01)00770-5

[CR6] AndreouT, Williams J, Brownlie RJ, Salmond RJ, Watson E, Shaw G, et al. Hematopoietic stem cell gene therapy targeting TGFβ enhances the efficacy of irradiation therapy in a preclinical glioblastoma model. J Immunother Cancer. 2021;9(3). 10.1136/jitc-2020-001143. 10.1136/jitc-2020-001143 PMC7957127 33707311

[CR7] Apostolou E, Blau H, Chien K, Lancaster MA, Tata PR, Trompouki E, et al. Progress and challenges in stem cell biology. Nat Cell Biol. 2023;25(2):203–6. 10.1038/s41556-023-01087-y. 36788378 10.1038/s41556-023-01087-y

[CR8] Atiya H, Frisbie L, Pressimone C, Coffman L. Mesenchymal stem cells in the tumor microenvironment. Tumor microenvironment: non-hematopoietic cells a birbrair. Cham: Springer International Publishing; 2020. p. 31–42. 10.1007/978-3-030-37184-5_3 32040853

[CR9] Attisano L, Wrana JL. Signal transduction by the TGF-beta superfamily. Science. 2002;296(5573):1646–7. 10.1126/science.1071809. 12040180 10.1126/science.1071809

[CR10] Avery S, Zafarana G, Gokhale PJ, Andrews PW. The role of SMAD4 in human embryonic stem cell self-renewal and stem cell fate. Stem Cells. 2010;28(5):863–73. 10.1002/stem.409. 20235236 10.1002/stem.409

[CR11] Ayob AZ, Ramasamy TS. Cancer stem cells as key drivers of tumour progression. J Biomed Sci. 2018;25(1):20. 10.1186/s12929-018-0426-4. 29506506 10.1186/s12929-018-0426-4 PMC5838954

[CR12] Baker CL, Pera MF. Capturing totipotent stem cells. Cell Stem Cell. 2018;22(1):25–34. 10.1016/j.stem.2017.12.011. 29304340 10.1016/j.stem.2017.12.011

[CR13] Bao S, Wu Q, McLendon RE, Hao Y, Shi Q, Hjelmeland AB, et al. Glioma stem cells promote radioresistance by preferential activation of the DNA damage response. Nature. 2006;444(7120):756–60. 10.1038/nature05236. 17051156 10.1038/nature05236

[CR14] Barker N, van Es JH, Kuipers J, Kujala P, van den Born M, Cozijnsen M, et al. Identification of stem cells in small intestine and colon by marker gene Lgr5. Nature. 2007;449(7165):1003–7. 10.1038/nature06196. 17934449 10.1038/nature06196

[CR15] Batlle R, Andrés E, Gonzalez L, Llonch E, Igea A, Gutierrez-Prat N, et al. Regulation of tumor angiogenesis and mesenchymal-endothelial transition by p38α through TGF-β and JNK signaling. Nat Commun. 2019;10(1):3071. 10.1038/s41467-019-10946-y. 31296856 10.1038/s41467-019-10946-y PMC6624205

[CR16] Bendall SC, Stewart MH, Menendez P, George D, Vijayaragavan K, Werbowetski-Ogilvie T, et al. IGF and FGF cooperatively establish the regulatory stem cell niche of pluripotent human cells in vitro. Nature. 2007;448(7157):1015–21. 10.1038/nature06027. 17625568 10.1038/nature06027

[CR17] Beyer TA, Narimatsu M, Weiss A, David L, Wrana JL. The TGFβ superfamily in stem cell biology and early mammalian embryonic development. Biochim Biophys Acta. 2013;1830(2):2268–79. 10.1016/j.bbagen.2012.08.025. 22967760 10.1016/j.bbagen.2012.08.025

[CR18] Bhatia M, Bonnet D, Wu D, Murdoch B, Wrana J, Gallacher L, et al. Bone morphogenetic proteins regulate the developmental program of human hematopoietic stem cells. J Exp Med. 1999;189(7):1139–48. 10.1084/jem.189.7.1139. 10190905 10.1084/jem.189.7.1139 PMC2193014

[CR19] Blank U, Karlsson S. TGF-β signaling in the control of hematopoietic stem cells. Blood. 2015;125(23):3542–50. 10.1182/blood-2014-12-618090. 25833962 10.1182/blood-2014-12-618090

[CR20] Boland GM, Perkins G, Hall DJ, Tuan RS. Wnt 3a promotes proliferation and suppresses osteogenic differentiation of adult human mesenchymal stem cells. J Cell Biochem. 2004;93(6):1210–30. 10.1002/jcb.20284. 15486964 10.1002/jcb.20284

[CR21] Bonaguidi MA, Peng CY, McGuire T, Falciglia G, Gobeske KT, Czeisler C, et al. Noggin expands neural stem cells in the adult hippocampus. J Neurosci. 2008;28(37):9194–204. 10.1523/jneurosci.3314-07.2008. 18784300 10.1523/JNEUROSCI.3314-07.2008 PMC3651371

[CR22] Botchkarev VA, Botchkareva NV, Nakamura M, Huber O, Funa K, Lauster R, et al. Noggin is required for induction of the hair follicle growth phase in postnatal skin. Faseb j. 2001;15(12):2205–14. 10.1096/fj.01-0207com. 11641247 10.1096/fj.01-0207com

[CR23] Boyer LA, Lee TI, Cole MF, Johnstone SE, Levine SS, Zucker JP, et al. Core transcriptional regulatory circuitry in human embryonic stem cells. Cell. 2005;122(6):947–56. 10.1016/j.cell.2005.08.020. 16153702 10.1016/j.cell.2005.08.020 PMC3006442

[CR24] Brown ML, Schneyer A. A decade later: revisiting the TGFβ family’s role in diabetes. Trends Endocrinol Metab. 2021;32(1):36–47. 10.1016/j.tem.2020.11.006. 33261990 10.1016/j.tem.2020.11.006

[CR25] Brown S, Teo A, Pauklin S, Hannan N, Cho CH, Lim B, et al. Activin/Nodal signaling controls divergent transcriptional networks in human embryonic stem cells and in endoderm progenitors. Stem Cells. 2011;29(8):1176–85. 10.1002/stem.666. 21630377 10.1002/stem.666

[CR26] Bryder D, Rossi DJ, Weissman IL. Hematopoietic stem cells: the paradigmatic tissue-specific stem cell. Am J Pathol. 2006;169(2):338–46. 10.2353/ajpath.2006.060312. 16877336 10.2353/ajpath.2006.060312 PMC1698791

[CR27] Buijs JT, van der Horst G, van den Hoogen C, Cheung H, de Rooij B, Kroon J, et al. The BMP2/7 heterodimer inhibits the human breast cancer stem cell subpopulation and bone metastases formation. Oncogene. 2012;31(17):2164–74. 10.1038/onc.2011.400. 21996751 10.1038/onc.2011.400

[CR28] Caja L, Kahata K, Moustakas A. Context-dependent action of transforming growth factor β family members on normal and cancer stem cells. Curr Pharm Des. 2012;18(27):4072–86. 10.2174/138161212802430459. 22630079 10.2174/138161212802430459

[CR29] Caja L, Bellomo C, Moustakas A. Transforming growth factor β and bone morphogenetic protein actions in brain tumors. FEBS Lett. 2015;589(14):1588–97. 10.1016/j.febslet.2015.04.058. 25957771 10.1016/j.febslet.2015.04.058

[CR30] Cao L, Shao M, Schilder J, Guise T, Mohammad KS, Matei D. Tissue transglutaminase links TGF-β, epithelial to mesenchymal transition and a stem cell phenotype in ovarian cancer. Oncogene. 2012;31(20):2521–34. 10.1038/onc.2011.429. 21963846 10.1038/onc.2011.429

[CR31] Caplan AI. Mesenchymal stem cells and gene therapy. Clin Orthop Relat Res. 2000;379 Suppl:S67-70. 10.1097/00003086-200010001-00010. 10.1097/00003086-200010001-00010 11039754

[CR32] Castiglioni A, Yang Y, Williams K, Gogineni A, Lane RS, Wang AW, et al. Combined PD-L1/TGFβ blockade allows expansion and differentiation of stem cell-like CD8 T cells in immune excluded tumors. Nat Commun. 2023;14(1):4703. 10.1038/s41467-023-40398-4. 37543621 10.1038/s41467-023-40398-4 PMC10404279

[CR33] Celià-Terrassa T, Jolly MK. Cancer stem cells and epithelial-to-mesenchymal transition in cancer metastasis. Cold Spring Harb Perspect Med. 2020;10(7):a036905. 10.1101/cshperspect.a036905. 31570380 10.1101/cshperspect.a036905 PMC7328448

[CR34] Chambers SM, Fasano CA, Papapetrou EP, Tomishima M, Sadelain M, Studer L. Highly efficient neural conversion of human ES and iPS cells by dual inhibition of SMAD signaling. Nat Biotechnol. 2009;27(3):275–80. 10.1038/nbt.1529. 19252484 10.1038/nbt.1529 PMC2756723

[CR35] Chen HL, Panchision DM. Concise review: bone morphogenetic protein pleiotropism in neural stem cells and their derivatives–alternative pathways, convergent signals. Stem Cells. 2007;25(1):63–8. 10.1634/stemcells.2006-0339. 16973830 10.1634/stemcells.2006-0339

[CR36] Chen X, Huang J, Wu J, Hao J, Fu B, Wang Y, et al. Human mesenchymal stem cells. Cell Prolif. 2022;55(4):e13141. 10.1111/cpr.13141. 34936710 10.1111/cpr.13141 PMC9055891

[CR37] Chen S-M, Guo B-J, Feng A-Q, Wang X-L, Zhang S-L, Miao C-Y. Pathways regulating intestinal stem cells and potential therapeutic targets for radiation enteropathy. Mol Biomed. 2024;5(1):46. 10.1186/s43556-024-00211-0. 39388072 10.1186/s43556-024-00211-0 PMC11467144

[CR38] Choy L, Skillington J, Derynck R. Roles of autocrine TGF-beta receptor and Smad signaling in adipocyte differentiation. J Cell Biol. 2000;149(3):667–82. 10.1083/jcb.149.3.667. 10791980 10.1083/jcb.149.3.667 PMC2174852

[CR39] Cotsarelis G, Sun TT, Lavker RM. Label-retaining cells reside in the bulge area of pilosebaceous unit: implications for follicular stem cells, hair cycle, and skin carcinogenesis. Cell. 1990;61(7):1329–37. 10.1016/0092-8674(90)90696-c. 2364430 10.1016/0092-8674(90)90696-c

[CR40] Creamer B, Shorter RG, Bamforth J. The turnover and shedding of epithelial cells. I. The turnover in the gastro-intestinal tract. Gut. 1961;2(2):110–8. 10.1136/gut.2.2.110.10.1136/gut.2.2.110. 13696345 10.1136/gut.2.2.110 PMC1413255

[CR41] Cristancho AG, Lazar MA. Forming functional fat: a growing understanding of adipocyte differentiation. Nat Rev Mol Cell Biol. 2011;12(11):722–34. 10.1038/nrm3198. 21952300 10.1038/nrm3198 PMC7171550

[CR42] D’Amour KA, Agulnick AD, Eliazer S, Kelly OG, Kroon E, Baetge EE. Efficient differentiation of human embryonic stem cells to definitive endoderm. Nat Biotechnol. 2005;23(12):1534–41. 10.1038/nbt1163. 16258519 10.1038/nbt1163

[CR43] Davis H, Irshad S, Bansal M, Rafferty H, Boitsova T, Bardella C, et al. Aberrant epithelial GREM1 expression initiates colonic tumorigenesis from cells outside the stem cell niche. Nat Med. 2015;21(1):62–70. 10.1038/nm.3750. 25419707 10.1038/nm.3750 PMC4594755

[CR44] De Luca M, Aiuti A, Cossu G, Parmar M, Pellegrini G, Robey PG. Advances in stem cell research and therapeutic development. Nat Cell Biol. 2019;21(7):801–11. 10.1038/s41556-019-0344-z. 31209293 10.1038/s41556-019-0344-z

[CR45] de Rooij DG. The nature and dynamics of spermatogonial stem cells. Development. 2017;144(17):3022–30. 10.1242/dev.146571. 28851723 10.1242/dev.146571

[CR46] Dewidar B, Meyer C, Dooley S, Meindl-Beinker AN. TGF-β in hepatic stellate cell activation and liver fibrogenesis-updated 2019. Cells. 2019;8(11):1419. 10.3390/cells8111419. 31718044 10.3390/cells8111419 PMC6912224

[CR47] Ding L, Saunders TL, Enikolopov G, Morrison SJ. Endothelial and perivascular cells maintain haematopoietic stem cells. Nature. 2012;481(7382):457–62. 10.1038/nature10783. 22281595 10.1038/nature10783 PMC3270376

[CR48] Dongre A, Weinberg RA. New insights into the mechanisms of epithelial-mesenchymal transition and implications for cancer. Nat Rev Mol Cell Biol. 2019;20(2):69–84. 10.1038/s41580-018-0080-4. 30459476 10.1038/s41580-018-0080-4

[CR49] Donovan PJ, Gearhart J. The end of the beginning for pluripotent stem cells. Nature. 2001;414(6859):92–7. 10.1038/35102154. 11689953 10.1038/35102154

[CR50] Du P, Wu J. Hallmarks of totipotent and pluripotent stem cell states. Cell Stem Cell. 2024;31(3):312–33. 10.1016/j.stem.2024.01.009. 38382531 10.1016/j.stem.2024.01.009 PMC10939785

[CR51] Duggal G, Heindryckx B, Warrier S, Taelman J, Van der Jeught M, Deforce D, et al. Exogenous supplementation of Activin A enhances germ cell differentiation of human embryonic stem cells. Mol Hum Reprod. 2015;21(5):410–23. 10.1093/molehr/gav004. 25634576 10.1093/molehr/gav004

[CR52] Dykstra B, Kent D, Bowie M, McCaffrey L, Hamilton M, Lyons K, et al. Long-term propagation of distinct hematopoietic differentiation programs in vivo. Cell Stem Cell. 2007;1(2):218–29. 10.1016/j.stem.2007.05.015. 18371352 10.1016/j.stem.2007.05.015

[CR53] Ehata S, Johansson E, Katayama R, Koike S, Watanabe A, Hoshino Y, et al. Transforming growth factor-β decreases the cancer-initiating cell population within diffuse-type gastric carcinoma cells. Oncogene. 2011;30(14):1693–705. 10.1038/onc.2010.546. 21132002 10.1038/onc.2010.546

[CR54] Essawy M, Shouman S, Magdy S, Abdelfattah-Hassan A, El-Badri N. Introduction and basic concepts in stem cell research and therapy: the facts and the hype. Regenerative medicine and stem cell biology. N. El-Badri. Cham: Springer International Publishing; 2020. p. 1–36. 10.1007/978-3-030-55359-3_1.

[CR55] Falk S, Wurdak H, Ittner LM, Ille F, Sumara G, Schmid MT, et al. Brain area-specific effect of TGF-beta signaling on Wnt-dependent neural stem cell expansion. Cell Stem Cell. 2008;2(5):472–83. 10.1016/j.stem.2008.03.006. 18462697 10.1016/j.stem.2008.03.006

[CR56] Fan W, Yuan H, Chang L, Li Q, Gao J, Ma L, et al. Role of the TGF-β signaling pathway in induced pluripotent stem cells reprogramming. Chin Med J (Engl). 2024;137(18):2263–5. 10.1097/cm9.0000000000003229. 39039625 10.1097/CM9.0000000000003229 PMC11407801

[CR57] Fei T, Chen YG. Regulation of embryonic stem cell self-renewal and differentiation by TGF-beta family signaling. Sci China Life Sci. 2010;53(4):497–503. 10.1007/s11427-010-0096-2. 20596917 10.1007/s11427-010-0096-2

[CR58] Ferraro F, Celso CL, Scadden D. Adult stem cels and their niches. Adv Exp Med Biol. 2010;695:155–68. 10.1007/978-1-4419-7037-4_11. 21222205 10.1007/978-1-4419-7037-4_11 PMC4020242

[CR59] Foitzik K, Paus R, Doetschman T, Dotto GP. The TGF-beta2 isoform is both a required and sufficient inducer of murine hair follicle morphogenesis. Dev Biol. 1999;212(2):278–89. 10.1006/dbio.1999.9325. 10433821 10.1006/dbio.1999.9325

[CR60] Foitzik K, Lindner G, Mueller-Roever S, Maurer M, Botchkareva N, Botchkarev V, et al. Control of murine hair follicle regression (catagen) by TGF-beta1 in vivo. Faseb j. 2000;14(5):752–60. 10.1096/fasebj.14.5.752. 10744631 10.1096/fasebj.14.5.752

[CR61] Fuchs E, Segre JA. Stem cells: a new lease on life. Cell. 2000;100(1):143–55. 10.1016/s0092-8674(00)81691-8. 10647939 10.1016/s0092-8674(00)81691-8

[CR62] Futakuchi M, Lami K, Tachibana Y, Yamamoto Y, Furukawa M, Fukuoka J. The Effects of TGF-β Signaling on Cancer Cells and Cancer Stem Cells in the Bone Microenvironment. Int J Mol Sci. 2019;20(20):5117. 10.3390/ijms20205117. 31619018 10.3390/ijms20205117 PMC6829436

[CR63] Gadue P, Huber TL, Paddison PJ, Keller GM. Wnt and TGF-beta signaling are required for the induction of an in vitro model of primitive streak formation using embryonic stem cells. Proc Natl Acad Sci U S A. 2006;103(45):16806–11. 10.1073/pnas.0603916103. 17077151 10.1073/pnas.0603916103 PMC1636536

[CR64] Gafni O, Weinberger L, Mansour AA, Manor YS, Chomsky E, Ben-Yosef D, et al. Derivation of novel human ground state naive pluripotent stem cells. Nature. 2013;504(7479):282–6. 10.1038/nature12745. 24172903 10.1038/nature12745

[CR65] Galvin-Burgess KE, Travis ED, Pierson KE, Vivian JL. TGF-β-superfamily signaling regulates embryonic stem cell heterogeneity: self-renewal as a dynamic and regulated equilibrium. Stem Cells. 2013;31(1):48–58. 10.1002/stem.1252. 23081664 10.1002/stem.1252 PMC3528825

[CR66] García-Gómez P, Golán I, S. D. M, A, Mezheyeuski A, Bellomo C, Tzavlaki K, et al. NOX4 regulates TGFβ-induced proliferation and self-renewal in glioblastoma stem cells. Mol Oncol. 2022;16(9):1891–912. 10.1002/1878-0261.13200. 35203105 10.1002/1878-0261.13200 PMC9067149

[CR67] Girardi F, Taleb A, Ebrahimi M, Datye A, Gamage DG, Peccate C, et al. TGFβ signaling curbs cell fusion and muscle regeneration. Nat Commun. 2021;12(1):750. 10.1038/s41467-020-20289-8. 33531466 10.1038/s41467-020-20289-8 PMC7854756

[CR68] Grafe I, Alexander S, Peterson JR, Snider TN, Levi B, Lee B, et al. TGF-β Family Signaling in Mesenchymal Differentiation. Cold Spring Harb Perspect Biol. 2018;10(5):a022202. 10.1101/cshperspect.a022202. 28507020 10.1101/cshperspect.a022202 PMC5932590

[CR69] Graham SM, Jørgensen HG, Allan E, Pearson C, Alcorn MJ, Richmond L, et al. Primitive, quiescent, Philadelphia-positive stem cells from patients with chronic myeloid leukemia are insensitive to STI571 in vitro. Blood. 2002;99(1):319–25. 10.1182/blood.v99.1.319. 11756187 10.1182/blood.v99.1.319

[CR70] Greber B, Wu G, Bernemann C, Joo JY, Han DW, Ko K, et al. Conserved and divergent roles of FGF signaling in mouse epiblast stem cells and human embryonic stem cells. Cell Stem Cell. 2010;6(3):215–26. 10.1016/j.stem.2010.01.003. 20207225 10.1016/j.stem.2010.01.003

[CR71] Greenbaum A, Hsu YM, Day RB, Schuettpelz LG, Christopher MJ, Borgerding JN, et al. CXCL12 in early mesenchymal progenitors is required for haematopoietic stem-cell maintenance. Nature. 2013;495(7440):227–30. 10.1038/nature11926. 23434756 10.1038/nature11926 PMC3600148

[CR72] Gregorieff A, Clevers H. Wnt signaling in the intestinal epithelium: from endoderm to cancer. Genes Dev. 2005;19(8):877–90. 10.1101/gad.1295405. 15833914 10.1101/gad.1295405

[CR73] Gregorieff A, Pinto D, Begthel H, Destrée O, Kielman M, Clevers H. Expression pattern of Wnt signaling components in the adult intestine. Gastroenterology. 2005;129(2):626–38. 10.1016/j.gastro.2005.06.007. 16083717 10.1016/j.gastro.2005.06.007

[CR74] Gross RE, Mehler MF, Mabie PC, Zang Z, Santschi L, Kessler JA. Bone morphogenetic proteins promote astroglial lineage commitment by mammalian subventricular zone progenitor cells. Neuron. 1996;17(4):595–606. 10.1016/s0896-6273(00)80193-2. 8893018 10.1016/s0896-6273(00)80193-2

[CR75] Gu S, Feng XH. TGF-β signaling in cancer. Acta Biochim Biophys Sin (Shanghai). 2018;50(10):941–9. 10.1093/abbs/gmy092. 30165534 10.1093/abbs/gmy092

[CR76] Guo L, Lin L, Wang X, Gao M, Cao S, Mai Y, et al. Resolving cell fate decisions during somatic cell reprogramming by single-cell RNA-Seq. Mol Cell. 2019;73(4):815-829.e817. 10.1016/j.molcel.2019.01.042. 30772174 10.1016/j.molcel.2019.01.042

[CR77] Haramis AP, Begthel H, van den Born M, van Es J, Jonkheer S, Offerhaus GJ, et al. De novo crypt formation and juvenile polyposis on BMP inhibition in mouse intestine. Science. 2004;303(5664):1684–6. 10.1126/science.1093587. 15017003 10.1126/science.1093587

[CR78] Hariyanto NI, Purwandhita RP, Syahrani RA, Louisa M, Wanandi SI. Role of TGF-β1 in human breast cancer stem cells. J Pak Med Assoc. 2021;71(Suppl 2)(2):S84-s89. 33785948

[CR79] Hata A, Chen YG. TGF-β signaling from receptors to smads. Cold Spring Harb Perspect Biol. 2016;8(9):a022061. 10.1101/cshperspect.a022061. 27449815 10.1101/cshperspect.a022061 PMC5008074

[CR80] He XC, Zhang J, Tong WG, Tawfik O, Ross J, Scoville DH, et al. BMP signaling inhibits intestinal stem cell self-renewal through suppression of Wnt-beta-catenin signaling. Nat Genet. 2004;36(10):1117–21. 10.1038/ng1430. 15378062 10.1038/ng1430

[CR81] He Y, Zhang H, Yung A, Villeda SA, Jaeger PA, Olayiwola O, et al. ALK5-dependent TGF-β signaling is a major determinant of late-stage adult neurogenesis. Nat Neurosci. 2014;17(7):943–52. 10.1038/nn.3732. 24859199 10.1038/nn.3732 PMC4096284

[CR82] Hoang DM, Pham PT, Bach TQ, Ngo ATL, Nguyen QT, Phan TTK, et al. Stem cell-based therapy for human diseases. Signal Transduct Target Ther. 2022;7(1):272. 10.1038/s41392-022-01134-4. 35933430 10.1038/s41392-022-01134-4 PMC9357075

[CR83] Hsu YC, Pasolli HA, Fuchs E. Dynamics between stem cells, niche, and progeny in the hair follicle. Cell. 2011;144(1):92–105. 10.1016/j.cell.2010.11.049. 21215372 10.1016/j.cell.2010.11.049 PMC3050564

[CR84] Huang T, Song X, Xu D, Tiek D, Goenka A, Wu B, et al. Stem cell programs in cancer initiation, progression, and therapy resistance. Theranostics. 2020;10(19):8721–43. 10.7150/thno.41648. 32754274 10.7150/thno.41648 PMC7392012

[CR85] Ichida JK, Blanchard J, Lam K, Son EY, Chung JE, Egli D, et al. A small-molecule inhibitor of tgf-Beta signaling replaces sox2 in reprogramming by inducing nanog. Cell Stem Cell. 2009;5(5):491–503. 10.1016/j.stem.2009.09.012. 19818703 10.1016/j.stem.2009.09.012 PMC3335195

[CR86] Ikushima H, Todo T, Ino Y, Takahashi M, Miyazawa K, Miyazono K. Autocrine TGF-beta signaling maintains tumorigenicity of glioma-initiating cells through Sry-related HMG-box factors. Cell Stem Cell. 2009;5(5):504–14. 10.1016/j.stem.2009.08.018. 19896441 10.1016/j.stem.2009.08.018

[CR87] Inman GJ, Nicolás FJ, Callahan JF, Harling JD, Gaster LM, Reith AD, et al. SB-431542 is a potent and specific inhibitor of transforming growth factor-beta superfamily type I activin receptor-like kinase (ALK) receptors ALK4, ALK5, and ALK7. Mol Pharmacol. 2002;62(1):65–74. 10.1124/mol.62.1.65. 12065756 10.1124/mol.62.1.65

[CR88] Itoh F, Watabe T, Miyazono K. Roles of TGF-β family signals in the fate determination of pluripotent stem cells. Semin Cell Dev Biol. 2014;32:98–106. 10.1016/j.semcdb.2014.05.017. 24910449 10.1016/j.semcdb.2014.05.017

[CR89] James D, Levine AJ, Besser D, Hemmati-Brivanlou A. TGFbeta/activin/nodal signaling is necessary for the maintenance of pluripotency in human embryonic stem cells. Development. 2005;132(6):1273–82. 10.1242/dev.01706. 15703277 10.1242/dev.01706

[CR90] Jian H, Shen X, Liu I, Semenov M, He X, Wang XF. Smad3-dependent nuclear translocation of beta-catenin is required for TGF-beta1-induced proliferation of bone marrow-derived adult human mesenchymal stem cells. Genes Dev. 2006;20(6):666–74. 10.1101/gad.1388806. 16543220 10.1101/gad.1388806 PMC1413283

[CR91] Jin W, Takagi T, Kanesashi SN, Kurahashi T, Nomura T, Harada J, et al. Schnurri-2 controls BMP-dependent adipogenesis via interaction with Smad proteins. Dev Cell. 2006;10(4):461–71. 10.1016/j.devcel.2006.02.016. 16580992 10.1016/j.devcel.2006.02.016

[CR92] Jørgensen A, Macdonald J, Nielsen JE, Kilcoyne KR, Perlman S, Lundvall L, et al. Nodal signaling regulates germ cell development and establishment of seminiferous cords in the human fetal testis. Cell Rep. 2018;25(7):1924-1937.e1924. 10.1016/j.celrep.2018.10.064. 30428358 10.1016/j.celrep.2018.10.064

[CR93] Jotzu C, Alt E, Welte G, Li J, Hennessy BT, Devarajan E, et al. Adipose tissue-derived stem cells differentiate into carcinoma-associated fibroblast-like cells under the influence of tumor-derived factors. Anal Cell Pathol (Amst). 2010;33(2):61–79. 10.3233/acp-clo-2010-0535. 20978328 10.3233/ACP-CLO-2010-0535 PMC4605656

[CR94] Kale VP, Vaidya AA. Molecular mechanisms behind the dose-dependent differential activation of MAPK pathways induced by transforming growth factor-beta1 in hematopoietic cells. Stem Cells Dev. 2004;13(5):536–47. 10.1089/scd.2004.13.536. 15588511 10.1089/scd.2004.13.536

[CR95] Kandasamy M, Lehner B, Kraus S, Sander PR, Marschallinger J, Rivera FJ, et al. TGF-beta signalling in the adult neurogenic niche promotes stem cell quiescence as well as generation of new neurons. J Cell Mol Med. 2014;18(7):1444–59. 10.1111/jcmm.12298. 24779367 10.1111/jcmm.12298 PMC4124027

[CR96] Kang Q, Sun MH, Cheng H, Peng Y, Montag AG, Deyrup AT, et al. Characterization of the distinct orthotopic bone-forming activity of 14 BMPs using recombinant adenovirus-mediated gene delivery. Gene Ther. 2004;11(17):1312–20. 10.1038/sj.gt.3302298. 15269709 10.1038/sj.gt.3302298

[CR97] Karampetsou M, Vekrellis K, Melachroinou K. The promise of the TGF-β superfamily as a therapeutic target for Parkinson’s disease. Neurobiol Dis. 2022;171:105805. 10.1016/j.nbd.2022.105805. 35764291 10.1016/j.nbd.2022.105805

[CR98] Katsuno Y, Derynck R. Epithelial plasticity, epithelial-mesenchymal transition, and the TGF-β family. Dev Cell. 2021;56(6):726–46. 10.1016/j.devcel.2021.02.028. 33756119 10.1016/j.devcel.2021.02.028

[CR99] Katsuno Y, Ehata S, Yashiro M, Yanagihara K, Hirakawa K, Miyazono K. Coordinated expression of REG4 and aldehyde dehydrogenase 1 regulating tumourigenic capacity of diffuse-type gastric carcinoma-initiating cells is inhibited by TGF-β. J Pathol. 2012;228(3):391–404. 10.1002/path.4020. 22430847 10.1002/path.4020

[CR100] Katsuno Y, Lamouille S, Derynck R. TGF-β signaling and epithelial-mesenchymal transition in cancer progression. Curr Opin Oncol. 2013;25(1):76–84. 10.1097/CCO.0b013e32835b6371. 23197193 10.1097/CCO.0b013e32835b6371

[CR101] Kee K, Gonsalves JM, Clark AT, Pera RA. Bone morphogenetic proteins induce germ cell differentiation from human embryonic stem cells. Stem Cells Dev. 2006;15(6):831–7. 10.1089/scd.2006.15.831. 17253946 10.1089/scd.2006.15.831

[CR102] Kobayashi A, Okuda H, Xing F, Pandey PR, Watabe M, Hirota S, et al. Bone morphogenetic protein 7 in dormancy and metastasis of prostate cancer stem-like cells in bone. J Exp Med. 2011;208(13):2641–55. 10.1084/jem.20110840. 22124112 10.1084/jem.20110840 PMC3244043

[CR103] Kobielak K, Stokes N, de la Cruz J, Polak L, Fuchs E. Loss of a quiescent niche but not follicle stem cells in the absence of bone morphogenetic protein signaling. Proc Natl Acad Sci U S A. 2007;104(24):10063–8. 10.1073/pnas.0703004104. 17553962 10.1073/pnas.0703004104 PMC1888574

[CR104] Kosinski C, Li VS, Chan AS, Zhang J, Ho C, Tsui WY, et al. Gene expression patterns of human colon tops and basal crypts and BMP antagonists as intestinal stem cell niche factors. Proc Natl Acad Sci U S A. 2007;104(39):15418–23. 10.1073/pnas.0707210104. 17881565 10.1073/pnas.0707210104 PMC2000506

[CR105] Krieglstein K. TGF-β in Brain Disorders. TGF-β in Human Disease. A Moustakas and K. Miyazawa. Tokyo: Springer Japan; 2013. p. 391–412. 10.1007/978-4-431-54409-8_17.

[CR106] Kriegstein A, Alvarez-Buylla A. The glial nature of embryonic and adult neural stem cells. Annu Rev Neurosci. 2009;32:149–84. 10.1146/annurev.neuro.051508.135600. 19555289 10.1146/annurev.neuro.051508.135600 PMC3086722

[CR107] Kubo A, Shinozaki K, Shannon JM, Kouskoff V, Kennedy M, Woo S, et al. Development of definitive endoderm from embryonic stem cells in culture. Development. 2004;131(7):1651–62. 10.1242/dev.01044. 14998924 10.1242/dev.01044

[CR108] Kulessa H, Turk G, Hogan BL. Inhibition of Bmp signaling affects growth and differentiation in the anagen hair follicle. Embo j. 2000;19(24):6664–74. 10.1093/emboj/19.24.6664. 11118201 10.1093/emboj/19.24.6664 PMC305899

[CR109] Lane SW, Scadden DT, Gilliland DG. The leukemic stem cell niche: current concepts and therapeutic opportunities. Blood. 2009;114(6):1150–7. 10.1182/blood-2009-01-202606. 19401558 10.1182/blood-2009-01-202606 PMC2723012

[CR110] Larsson J, Karlsson S. The role of Smad signaling in hematopoiesis. Oncogene. 2005;24(37):5676–92. 10.1038/sj.onc.1208920. 16123801 10.1038/sj.onc.1208920

[CR111] Lasorella A, Benezra R, Iavarone A. The ID proteins: master regulators of cancer stem cells and tumour aggressiveness. Nat Rev Cancer. 2014;14(2):77–91. 10.1038/nrc3638. 24442143 10.1038/nrc3638

[CR112] Leblond CP, Stevens CE. The constant renewal of the intestinal epithelium in the albino rat. Anat Rec. 1948;100(3):357–77. 10.1002/ar.1091000306. 18906253 10.1002/ar.1091000306

[CR113] Lee KS, Kim HJ, Li QL, Chi XZ, Ueta C, Komori T, et al. Runx2 is a common target of transforming growth factor beta1 and bone morphogenetic protein 2, and cooperation between Runx2 and Smad5 induces osteoblast-specific gene expression in the pluripotent mesenchymal precursor cell line C2C12. Mol Cell Biol. 2000;20(23):8783–92. 10.1128/mcb.20.23.8783-8792.2000. 11073979 10.1128/mcb.20.23.8783-8792.2000 PMC86511

[CR114] Lee J, Son MJ, Woolard K, Donin NM, Li A, Cheng CH, et al. Epigenetic-mediated dysfunction of the bone morphogenetic protein pathway inhibits differentiation of glioblastoma-initiating cells. Cancer Cell. 2008;13(1):69–80. 10.1016/j.ccr.2007.12.005. 18167341 10.1016/j.ccr.2007.12.005 PMC2835498

[CR115] Lee SW, Min SO, Bak SY, Hwang HK, Kim KS. Efficient endodermal induction of human adipose stem cells using various concentrations of Activin A for hepatic differentiation. Biochem Biophys Res Commun. 2015;464(4):1178–84. 10.1016/j.bbrc.2015.07.100. 26208453 10.1016/j.bbrc.2015.07.100

[CR116] Lee JH, Lee JH, Rane SG. TGF-β Signaling in Pancreatic Islet β Cell Development and Function. Endocrinology. 2021;162(3):bqaa233. 10.1210/endocr/bqaa233. 33349851 10.1210/endocr/bqaa233 PMC8240135

[CR117] Li R, Liang J, Ni S, Zhou T, Qing X, Li H, et al. A mesenchymal-to-epithelial transition initiates and is required for the nuclear reprogramming of mouse fibroblasts. Cell Stem Cell. 2010;7(1):51–63. 10.1016/j.stem.2010.04.014. 20621050 10.1016/j.stem.2010.04.014

[CR118] Li Z, Yang CS, Nakashima K, Rana TM. Small RNA-mediated regulation of iPS cell generation. Embo j. 2011;30(5):823–34. 10.1038/emboj.2011.2. 21285944 10.1038/emboj.2011.2 PMC3049216

[CR119] Li GC, Zhang HW, Zhao QC, Sun LI, Yang JJ, Hong L, et al. Mesenchymal stem cells promote tumor angiogenesis via the action of transforming growth factor β1. Oncol Lett. 2016a;11(2):1089–94. 10.3892/ol.2015.3997. 26893697 10.3892/ol.2015.3997 PMC4733964

[CR120] Li W, Wei W, Ding S. TGF-β signaling in stem cell regulation. Methods Mol Biol. 2016b;1344:137–45. 10.1007/978-1-4939-2966-5_8. 26520122 10.1007/978-1-4939-2966-5_8

[CR121] Liang C, Liao L, Tian W. Stem cell-based dental pulp regeneration: insights from signaling pathways. Stem Cell Rev Rep. 2021;17(4):1251–63. 10.1007/s12015-020-10117-3. 33459973 10.1007/s12015-020-10117-3

[CR122] Lichtman MK, Otero-Vinas M, Falanga V. Transforming growth factor beta (TGF-β) isoforms in wound healing and fibrosis. Wound Repair Regen. 2016;24(2):215–22. 10.1111/wrr.12398. 26704519 10.1111/wrr.12398

[CR123] Lim DA, Tramontin AD, Trevejo JM, Herrera DG, García-Verdugo JM, Alvarez-Buylla A. Noggin antagonizes BMP signaling to create a niche for adult neurogenesis. Neuron. 2000;28(3):713–26. 10.1016/s0896-6273(00)00148-3. 11163261 10.1016/s0896-6273(00)00148-3

[CR124] Lin T, Ambasudhan R, Yuan X, Li W, Hilcove S, Abujarour R, et al. A chemical platform for improved induction of human iPSCs. Nat Methods. 2009;6(11):805–8. 10.1038/nmeth.1393. 19838168 10.1038/nmeth.1393 PMC3724527

[CR125] Liu Z, Tang Y, Qiu T, Cao X, Clemens TL. A dishevelled-1/Smad1 interaction couples WNT and bone morphogenetic protein signaling pathways in uncommitted bone marrow stromal cells. J Biol Chem. 2006;281(25):17156–63. 10.1074/jbc.M513812200. 16621789 10.1074/jbc.M513812200

[CR126] Liu R, Wang JH, Xu C, Sun B, Kang SO. Activin pathway enhances colorectal cancer stem cell self-renew and tumor progression. Biochem Biophys Res Commun. 2016;479(4):715–20. 10.1016/j.bbrc.2016.09.146. 27693580 10.1016/j.bbrc.2016.09.146

[CR127] Lombardo Y, Scopelliti A, Cammareri P, Todaro M, Iovino F, Ricci-Vitiani L, et al. Bone morphogenetic protein 4 induces differentiation of colorectal cancer stem cells and increases their response to chemotherapy in mice. Gastroenterology. 2011;140(1):297–309. 10.1053/j.gastro.2010.10.005. 20951698 10.1053/j.gastro.2010.10.005

[CR128] Lonardo E, Hermann PC, Mueller MT, Huber S, Balic A, Miranda-Lorenzo I, et al. Nodal/Activin signaling drives self-renewal and tumorigenicity of pancreatic cancer stem cells and provides a target for combined drug therapy. Cell Stem Cell. 2011;9(5):433–46. 10.1016/j.stem.2011.10.001. 22056140 10.1016/j.stem.2011.10.001

[CR129] Luu HH, Song WX, Luo X, Manning D, Luo J, Deng ZL, et al. Distinct roles of bone morphogenetic proteins in osteogenic differentiation of mesenchymal stem cells. J Orthop Res. 2007;25(5):665–77. 10.1002/jor.20359. 17290432 10.1002/jor.20359

[CR130] Madhu V, Dighe AS, Cui Q, Deal DN. Dual inhibition of activin/Nodal/TGF-β and BMP signaling pathways by SB431542 and dorsomorphin induces neuronal differentiation of human adipose derived stem cells. Stem Cells Int. 2016;2016:1035374. 10.1155/2016/1035374. 26798350 10.1155/2016/1035374 PMC4699250

[CR131] Maeda S, Hayashi M, Komiya S, Imamura T, Miyazono K. Endogenous TGF-beta signaling suppresses maturation of osteoblastic mesenchymal cells. Embo j. 2004;23(3):552–63. 10.1038/sj.emboj.7600067. 14749725 10.1038/sj.emboj.7600067 PMC1271802

[CR132] Maherali N, Hochedlinger K. Guidelines and techniques for the generation of induced pluripotent stem cells. Cell Stem Cell. 2008;3(6):595–605. 10.1016/j.stem.2008.11.008. 19041776 10.1016/j.stem.2008.11.008

[CR133] Maherali N, Hochedlinger K. Tgfbeta signal inhibition cooperates in the induction of iPSCs and replaces Sox2 and cMyc. Curr Biol. 2009;19(20):1718–23. 10.1016/j.cub.2009.08.025. 19765992 10.1016/j.cub.2009.08.025 PMC3538372

[CR134] Mahmoudi R, Afshar S, Amini R, Jalali A, Saidijam M, Najafi R. Evaluation of BMP-2 as a differentiating and radiosensitizing agent for colorectal cancer stem cells. Curr Stem Cell Res Ther. 2024;19(1):83–93. 10.2174/1574888x18666230330085615. 36998132 10.2174/1574888X18666230330085615

[CR135] Marshall GP 2nd, Laywell ED, Zheng T, Steindler DA, Scott EW. In vitro-derived “neural stem cells” function as neural progenitors without the capacity for self-renewal. Stem Cells. 2006;24(3):731–8. 10.1634/stemcells.2005-0245. 16339644 10.1634/stemcells.2005-0245

[CR136] Massagué J, Sheppard D. TGF-β signaling in health and disease. Cell. 2023;186(19):4007–37. 10.1016/j.cell.2023.07.036. 37714133 10.1016/j.cell.2023.07.036 PMC10772989

[CR137] Massagué J, Xi Q. TGF-β control of stem cell differentiation genes. FEBS Lett. 2012;586(14):1953–8. 10.1016/j.febslet.2012.03.023. 22710171 10.1016/j.febslet.2012.03.023 PMC3466472

[CR138] Matsumoto T, Yokoi A, Hashimura M, Oguri Y, Akiya M, Saegusa M. TGF-β-mediated LEFTY/Akt/GSK-3β/Snail axis modulates epithelial-mesenchymal transition and cancer stem cell properties in ovarian clear cell carcinomas. Mol Carcinog. 2018;57(8):957–67. 10.1002/mc.22816. 29603383 10.1002/mc.22816

[CR139] Mazzella M, Walker K, Cormier C, Kapanowski M, Ishmakej A, Saifee A, et al. Regulation of self-renewal and senescence in primitive mesenchymal stem cells by Wnt and TGFβ signaling. Stem Cell Res Ther. 2023;14(1):305. 10.1186/s13287-023-03533-y. 37880755 10.1186/s13287-023-03533-y PMC10601332

[CR140] Mesa KR, Rompolas P, Zito G, Myung P, Sun TY, Brown S, et al. Niche-induced cell death and epithelial phagocytosis regulate hair follicle stem cell pool. Nature. 2015;522(7554):94–7. 10.1038/nature14306. 25849774 10.1038/nature14306 PMC4457634

[CR141] Meyers EA, Kessler JA. TGF-β Family Signaling in Neural and Neuronal Differentiation, Development, and Function. Cold Spring Harb Perspect Biol. 2017;9(8):a022244. 10.1101/cshperspect.a022244. 28130363 10.1101/cshperspect.a022244 PMC5538418

[CR142] Miettinen PJ, Ebner R, Lopez AR, Derynck R. TGF-beta induced transdifferentiation of mammary epithelial cells to mesenchymal cells: involvement of type I receptors. J Cell Biol. 1994;127(6 Pt 2):2021–36. 10.1083/jcb.127.6.2021. 7806579 10.1083/jcb.127.6.2021 PMC2120317

[CR143] Millar SE. Molecular mechanisms regulating hair follicle development. J Invest Dermatol. 2002;118(2):216–25. 10.1046/j.0022-202x.2001.01670.x. 11841536 10.1046/j.0022-202x.2001.01670.x

[CR144] Mishra S, Taelman J, Popovic M, Tilleman L, Duthoo E, van der Jeught M, et al. Activin A-derived human embryonic stem cells show increased competence to differentiate into primordial germ cell-like cells. Stem Cells. 2021;39(5):551–63. 10.1002/stem.3335. 33470497 10.1002/stem.3335 PMC8248136

[CR145] Miyoshi N, Ishii H, Nagano H, Haraguchi N, Dewi DL, Kano Y, et al. Reprogramming of mouse and human cells to pluripotency using mature microRNAs. Cell Stem Cell. 2011;8(6):633–8. 10.1016/j.stem.2011.05.001. 21620789 10.1016/j.stem.2011.05.001

[CR146] Moradi S, Mahdizadeh H, Šarić T, Kim J, Harati J, Shahsavarani H, et al. Research and therapy with induced pluripotent stem cells (iPSCs): social, legal, and ethical considerations. Stem Cell Res Ther. 2019;10(1):341. 10.1186/s13287-019-1455-y. 31753034 10.1186/s13287-019-1455-y PMC6873767

[CR147] Morizane A, Doi D, Kikuchi T, Nishimura K, Takahashi J. Small-molecule inhibitors of bone morphogenic protein and activin/nodal signals promote highly efficient neural induction from human pluripotent stem cells. J Neurosci Res. 2011;89(2):117–26. 10.1002/jnr.22547. 21162120 10.1002/jnr.22547

[CR148] Morrison SJ, Scadden DT. The bone marrow niche for haematopoietic stem cells. Nature. 2014;505(7483):327–34. 10.1038/nature12984. 24429631 10.1038/nature12984 PMC4514480

[CR149] Moses HL, Roberts AB, Derynck R. The discovery and early days of TGF-β: a historical perspective. Cold Spring Harb Perspect Biol. 2016;8(7):a021865. 10.1101/cshperspect.a021865. 27328871 10.1101/cshperspect.a021865 PMC4930926

[CR150] Moshaverinia A, Xu X, Chen C, Ansari S, Zadeh HH, Snead ML, et al. Application of stem cells derived from the periodontal ligament or gingival tissue sources for tendon tissue regeneration. Biomaterials. 2014;35(9):2642–50. 10.1016/j.biomaterials.2013.12.053. 24397989 10.1016/j.biomaterials.2013.12.053 PMC3929697

[CR151] Mousaei Ghasroldasht M, Seok J, Park HS, Liakath Ali FB, Al-Hendy A. Stem cell therapy: from idea to clinical practice. Int J Mol Sci. 2022;23(5):2850. 10.3390/ijms23052850. 35269990 10.3390/ijms23052850 PMC8911494

[CR152] Mullen AC, Wrana JL. TGF-β family signaling in embryonic and somatic stem-cell renewal and differentiation. Cold Spring Harb Perspect Biol. 2017;9(7):a022186. 10.1101/cshperspect.a022186. 28108485 10.1101/cshperspect.a022186 PMC5495062

[CR153] Naka K, Hoshii T, Muraguchi T, Tadokoro Y, Ooshio T, Kondo Y, et al. TGF-beta-FOXO signalling maintains leukaemia-initiating cells in chronic myeloid leukaemia. Nature. 2010;463(7281):676–80. 10.1038/nature08734. 20130650 10.1038/nature08734

[CR154] Nakao A, Afrakhte M, Morn A, Nakayama T, Christian JL, Heuchel R, et al. Identification of Smad7, a TGFβ-inducible antagonist of TGF-β signalling. Nature. 1997;389(6651):631–5. 10.1038/39369. 9335507 10.1038/39369

[CR155] Narushima Y, Kozuka-Hata H, Koyama-Nasu R, Tsumoto K, Inoue J, Akiyama T, et al. Integrative Network Analysis Combined with Quantitative Phosphoproteomics Reveals Transforming Growth Factor-beta Receptor type-2 (TGFBR2) as a Novel Regulator of Glioblastoma Stem Cell Properties. Mol Cell Proteomics. 2016;15(3):1017–31. 10.1074/mcp.M115.049999. 26670566 10.1074/mcp.M115.049999 PMC4813685

[CR156] Niola F, Zhao X, Singh D, Castano A, Sullivan R, Lauria M, et al. Id proteins synchronize stemness and anchorage to the niche of neural stem cells. Nat Cell Biol. 2012;14(5):477–87. 10.1038/ncb2490. 22522171 10.1038/ncb2490 PMC3635493

[CR157] Nishikawa S, Goldstein RA, Nierras CR. The promise of human induced pluripotent stem cells for research and therapy. Nat Rev Mol Cell Biol. 2008;9(9):725–9. 10.1038/nrm2466. 18698329 10.1038/nrm2466

[CR158] Nishimura EK, Suzuki M, Igras V, Du J, Lonning S, Miyachi Y, et al. Key roles for transforming growth factor beta in melanocyte stem cell maintenance. Cell Stem Cell. 2010;6(2):130–40. 10.1016/j.stem.2009.12.010. 20144786 10.1016/j.stem.2009.12.010 PMC3437996

[CR159] Nostro MC, Cheng X, Keller GM, Gadue P. Wnt, activin, and BMP signaling regulate distinct stages in the developmental pathway from embryonic stem cells to blood. Cell Stem Cell. 2008;2(1):60–71. 10.1016/j.stem.2007.10.011. 18371422 10.1016/j.stem.2007.10.011 PMC2533280

[CR160] Nunes T, Hamdan D, Leboeuf C, El Bouchtaoui M, Gapihan G, Nguyen TT, et al. Targeting cancer stem cells to overcome chemoresistance. Int J Mol Sci. 2018;19(12):4036. 10.3390/ijms19124036. 30551640 10.3390/ijms19124036 PMC6321478

[CR161] O’Brien CA, Kreso A, Ryan P, Hermans KG, Gibson L, Wang Y, et al. ID1 and ID3 regulate the self-renewal capacity of human colon cancer-initiating cells through p21. Cancer Cell. 2012;21(6):777–92. 10.1016/j.ccr.2012.04.036. 22698403 10.1016/j.ccr.2012.04.036

[CR162] Oshimori N, Fuchs E. Paracrine TGF-β signaling counterbalances BMP-mediated repression in hair follicle stem cell activation. Cell Stem Cell. 2012;10(1):63–75. 10.1016/j.stem.2011.11.005. 22226356 10.1016/j.stem.2011.11.005 PMC3349223

[CR163] Osnato A, Brown S, Krueger C, Andrews S, Collier AJ, Nakanoh S, et al. TGFβ signalling is required to maintain pluripotency of human naïve pluripotent stem cells. Elife. 2021;10:e67259. 10.7554/eLife.67259. 34463252 10.7554/eLife.67259 PMC8410071

[CR164] Panchision DM, Pickel JM, Studer L, Lee SH, Turner PA, Hazel TG, et al. Sequential actions of BMP receptors control neural precursor cell production and fate. Genes Dev. 2001;15(16):2094–110. 10.1101/gad.894701. 11511541 10.1101/gad.894701 PMC312756

[CR165] Panula S, Medrano JV, Kee K, Bergström R, Nguyen HN, Byers B, et al. Human germ cell differentiation from fetal- and adult-derived induced pluripotent stem cells. Hum Mol Genet. 2011;20(4):752–62. 10.1093/hmg/ddq520. 21131292 10.1093/hmg/ddq520 PMC3024045

[CR166] Parashurama N, Nahmias Y, Cho CH, van Poll D, Tilles AW, Berthiaume F, et al. Activin alters the kinetics of endoderm induction in embryonic stem cells cultured on collagen gels. Stem Cells. 2007;26(2):474–84. 10.1634/stemcells.2007-0303. 18065398 10.1634/stemcells.2007-0303 PMC2802581

[CR167] Park C, Afrikanova I, Chung YS, Zhang WJ, Arentson E, Fong GH, et al. A hierarchical order of factors in the generation of FLK1- and SCL-expressing hematopoietic and endothelial progenitors from embryonic stem cells. Development. 2004;131(11):2749–62. 10.1242/dev.01130. 15148304 10.1242/dev.01130

[CR168] Pauklin S, Vallier L. Activin/Nodal signalling in stem cells. Development. 2015;142(4):607–19. 10.1242/dev.091769. 25670788 10.1242/dev.091769

[CR169] Peñuelas S, Anido J, Prieto-Sánchez RM, Folch G, Barba I, Cuartas I, et al. TGF-beta increases glioma-initiating cell self-renewal through the induction of LIF in human glioblastoma. Cancer Cell. 2009;15(4):315–27. 10.1016/j.ccr.2009.02.011. 19345330 10.1016/j.ccr.2009.02.011

[CR170] Pera MF, Andrade J, Houssami S, Reubinoff B, Trounson A, Stanley EG, et al. Regulation of human embryonic stem cell differentiation by BMP-2 and its antagonist noggin. J Cell Sci. 2004;117(Pt 7):1269–80. 10.1242/jcs.00970. 14996946 10.1242/jcs.00970

[CR171] Phinney DG, Prockop DJ. Concise review: mesenchymal stem/multipotent stromal cells: the state of transdifferentiation and modes of tissue repair–current views. Stem Cells. 2007;25(11):2896–902. 10.1634/stemcells.2007-0637. 17901396 10.1634/stemcells.2007-0637

[CR172] Piccirillo SG, Reynolds BA, Zanetti N, Lamorte G, Binda E, Broggi G, et al. Bone morphogenetic proteins inhibit the tumorigenic potential of human brain tumour-initiating cells. Nature. 2006;444(7120):761–5. 10.1038/nature05349. 17151667 10.1038/nature05349

[CR173] Pittenger MF, Mackay AM, Beck SC, Jaiswal RK, Douglas R, Mosca JD, et al. Multilineage potential of adult human mesenchymal stem cells. Science. 1999;284(5411):143–7. 10.1126/science.284.5411.143. 10102814 10.1126/science.284.5411.143

[CR174] Plikus MV, Mayer JA, de la Cruz D, Baker RE, Maini PK, Maxson R, et al. Cyclic dermal BMP signalling regulates stem cell activation during hair regeneration. Nature. 2008;451(7176):340–4. 10.1038/nature06457. 18202659 10.1038/nature06457 PMC2696201

[CR175] Ploemacher RE, Engels LJ, Mayer AE, Thies S, Neben S. Bone morphogenetic protein 9 is a potent synergistic factor for murine hemopoietic progenitor cell generation and colony formation in serum-free cultures. Leukemia. 1999;13(3):428–37. 10.1038/sj.leu.2401363. 10086734 10.1038/sj.leu.2401363

[CR176] Popova AP, Bozyk PD, Goldsmith AM, Linn MJ, Lei J, Bentley JK, et al. Autocrine production of TGF-beta1 promotes myofibroblastic differentiation of neonatal lung mesenchymal stem cells. Am J Physiol Lung Cell Mol Physiol. 2010;298(6):L735-743. 10.1152/ajplung.00347.2009. 20190033 10.1152/ajplung.00347.2009 PMC2886615

[CR177] Qi Y, Jiang D, Sindrilaru A, Stegemann A, Schatz S, Treiber N, et al. TSG-6 released from intradermally injected mesenchymal stem cells accelerates wound healing and reduces tissue fibrosis in murine full-thickness skin wounds. J Invest Dermatol. 2014;134(2):526–37. 10.1038/jid.2013.328. 23921952 10.1038/jid.2013.328

[CR178] Qin L, Liu N, Bao CL, Yang DZ, Ma GX, Yi WH, et al. Mesenchymal stem cells in fibrotic diseases—the two sides of the same coin. Acta Pharmacol Sin. 2023;44(2):268–87. 10.1038/s41401-022-00952-0. 35896695 10.1038/s41401-022-00952-0 PMC9326421

[CR179] Quéré R, Karlsson G, Hertwig F, Rissler M, Lindqvist B, Fioretos T, et al. Smad4 binds Hoxa9 in the cytoplasm and protects primitive hematopoietic cells against nuclear activation by Hoxa9 and leukemia transformation. Blood. 2011;117(22):5918–30. 10.1182/blood-2010-08-301879. 21471525 10.1182/blood-2010-08-301879

[CR180] Reya T, Morrison SJ, Clarke MF, Weissman IL. Stem cells, cancer, and cancer stem cells. Nature. 2001;414(6859):105–11. 10.1038/35102167. 11689955 10.1038/35102167

[CR181] Reynolds BA, Weiss S. Generation of neurons and astrocytes from isolated cells of the adult mammalian central nervous system. Science. 1992;255(5052):1707–10. 10.1126/science.1553558. 1553558 10.1126/science.1553558

[CR182] Robinton DA, Daley GQ. The promise of induced pluripotent stem cells in research and therapy. Nature. 2012;481(7381):295–305. 10.1038/nature10761. 22258608 10.1038/nature10761 PMC3652331

[CR183] Roelen BA, Dijke P. Controlling mesenchymal stem cell differentiation by TGFBeta family members. J Orthop Sci. 2003;8(5):740–8. 10.1007/s00776-003-0702-2. 14557946 10.1007/s00776-003-0702-2

[CR184] Ruscetti FW, Akel S, Bartelmez SH. Autocrine transforming growth factor-beta regulation of hematopoiesis: many outcomes that depend on the context. Oncogene. 2005;24(37):5751–63. 10.1038/sj.onc.1208921. 16123808 10.1038/sj.onc.1208921

[CR185] Sachdeva R, Wu M, Johnson K, Kim H, Celebre A, Shahzad U, et al. BMP signaling mediates glioma stem cell quiescence and confers treatment resistance in glioblastoma. Sci Rep. 2019;9(1):14569. 10.1038/s41598-019-51270-1. 31602000 10.1038/s41598-019-51270-1 PMC6787003

[CR186] Sakaki-Yumoto M, Katsuno Y, Derynck R. TGF-β family signaling in stem cells. Biochim Biophys Acta. 2013a;1830(2):2280–96. 10.1016/j.bbagen.2012.08.008. 22959078 10.1016/j.bbagen.2012.08.008 PMC4240309

[CR187] Sakaki-Yumoto M, Liu J, Ramalho-Santos M, Yoshida N, Derynck R. Smad2 is essential for maintenance of the human and mouse primed pluripotent stem cell state. J Biol Chem. 2013b;288(25):18546–60. 10.1074/jbc.M112.446591. 23649632 10.1074/jbc.M112.446591 PMC3689995

[CR188] Samavarchi-Tehrani P, Golipour A, David L, Sung HK, Beyer TA, Datti A, et al. Functional genomics reveals a BMP-driven mesenchymal-to-epithelial transition in the initiation of somatic cell reprogramming. Cell Stem Cell. 2010;7(1):64–77. 10.1016/j.stem.2010.04.015. 20621051 10.1016/j.stem.2010.04.015

[CR189] Sánchez-Duffhues G, Hiepen C. Human iPSCs as model systems for BMP-related rare diseases. Cells. 2023;12(17):2200. 10.3390/cells12172200. 37681932 10.3390/cells12172200 PMC10487005

[CR190] Sangiorgi E, Capecchi MR. Bmi1 is expressed in vivo in intestinal stem cells. Nat Genet. 2008;40(7):915–20. 10.1038/ng.165. 18536716 10.1038/ng.165 PMC2906135

[CR191] Scheel C, Eaton EN, Li SH, Chaffer CL, Reinhardt F, Kah KJ, et al. Paracrine and autocrine signals induce and maintain mesenchymal and stem cell states in the breast. Cell. 2011;145(6):926–40. 10.1016/j.cell.2011.04.029. 21663795 10.1016/j.cell.2011.04.029 PMC3930331

[CR192] Schmidt-Ullrich R, Paus R. Molecular principles of hair follicle induction and morphogenesis. BioEssays. 2005;27(3):247–61. 10.1002/bies.20184. 15714560 10.1002/bies.20184

[CR193] Schober M, Fuchs E. Tumor-initiating stem cells of squamous cell carcinomas and their control by TGF-β and integrin/focal adhesion kinase (FAK) signaling. Proc Natl Acad Sci U S A. 2011;108(26):10544–9. 10.1073/pnas.1107807108. 21670270 10.1073/pnas.1107807108 PMC3127891

[CR194] Schulz TC, Noggle SA, Palmarini GM, Weiler DA, Lyons IG, Pensa KA, et al. Differentiation of human embryonic stem cells to dopaminergic neurons in serum-free suspension culture. Stem Cells. 2004;22(7):1218–38. 10.1634/stemcells.2004-0114. 15579641 10.1634/stemcells.2004-0114

[CR195] Seuntjens E, Umans L, Zwijsen A, Sampaolesi M, Verfaillie CM, Huylebroeck D. Transforming Growth Factor type beta and Smad family signaling in stem cell function. Cytokine Growth Factor Rev. 2009;20(5–6):449–58. 10.1016/j.cytogfr.2009.10.005. 19892581 10.1016/j.cytogfr.2009.10.005

[CR196] Shah AA, Khan FA. Types and classification of stem cells. Advances in application of stem cells: from bench to clinic. FA Khan. Cham: Springer International Publishing; 2021. p. 25–49. 10.1007/978-3-030-78101-9_2.

[CR197] Shaim H, Shanley M, Basar R, Daher M, Gumin J, Zamler DB, et al. Targeting the αv integrin/TGF-β axis improves natural killer cell function against glioblastoma stem cells. J Clin Invest. 2021;131(14):e142116. 10.1172/JCI142116. 10.1172/JCI142116 PMC8279586 34138753

[CR198] Shi S, Bartold PM, Miura M, Seo BM, Robey PG, Gronthos S. The efficacy of mesenchymal stem cells to regenerate and repair dental structures. Orthod Craniofac Res. 2005;8(3):191–9. 10.1111/j.1601-6343.2005.00331.x. 16022721 10.1111/j.1601-6343.2005.00331.x

[CR199] Snippert HJ, Clevers H. Tracking adult stem cells. EMBO Rep. 2011;12(2):113–22. 10.1038/embor.2010.216. 21252944 10.1038/embor.2010.216 PMC3049439

[CR200] Söderberg SS, Karlsson G, Karlsson S. Complex and context dependent regulation of hematopoiesis by TGF-beta superfamily signaling. Ann N Y Acad Sci. 2009;1176:55–69. 10.1111/j.1749-6632.2009.04569.x. 19796233 10.1111/j.1749-6632.2009.04569.x

[CR201] Soma T, Dohrmann CE, Hibino T, Raftery LA. Profile of transforming growth factor-beta responses during the murine hair cycle. J Invest Dermatol. 2003;121(5):969–75. 10.1046/j.1523-1747.2003.12516.x. 14708594 10.1046/j.1523-1747.2003.12516.x

[CR202] Spradling A, Drummond-Barbosa D, Kai T. Stem cells find their niche. Nature. 2001;414(6859):98–104. 10.1038/35102160. 11689954 10.1038/35102160

[CR203] Stewart A, Guan H, Yang K. BMP-3 promotes mesenchymal stem cell proliferation through the TGF-beta/activin signaling pathway. J Cell Physiol. 2010;223(3):658–66. 10.1002/jcp.22064. 20143330 10.1002/jcp.22064

[CR204] Subramanyam D, Lamouille S, Judson RL, Liu JY, Bucay N, Derynck R, et al. Multiple targets of miR-302 and miR-372 promote reprogramming of human fibroblasts to induced pluripotent stem cells. Nat Biotechnol. 2011;29(5):443–8. 10.1038/nbt.1862. 21490602 10.1038/nbt.1862 PMC3685579

[CR205] Sugiyama T, Kohara H, Noda M, Nagasawa T. Maintenance of the hematopoietic stem cell pool by CXCL12-CXCR4 chemokine signaling in bone marrow stromal cell niches. Immunity. 2006;25(6):977–88. 10.1016/j.immuni.2006.10.016. 17174120 10.1016/j.immuni.2006.10.016

[CR206] Suzuki A, Raya Á, Kawakami Y, Morita M, Matsui T, Nakashima K, et al. Nanog binds to Smad1 and blocks bone morphogenetic protein-induced differentiation of embryonic stem cells. Proc Natl Acad Sci U S A. 2006;103(27):10294–9. 10.1073/pnas.0506945103. 16801560 10.1073/pnas.0506945103 PMC1502451

[CR207] Suzuki T, Lee CH, Chen M, Zhao W, Fu SY, Qi JJ, et al. Induced migration of dental pulp stem cells for in vivo pulp regeneration. J Dent Res. 2011;90(8):1013–8. 10.1177/0022034511408426. 21586666 10.1177/0022034511408426

[CR208] Taherian M, Bayati P, Mojtabavi N. Stem cell-based therapy for fibrotic diseases: mechanisms and pathways. Stem Cell Res Ther. 2024;15(1):170. 10.1186/s13287-024-03782-5. 38886859 10.1186/s13287-024-03782-5 PMC11184790

[CR209] Takahashi K, Tanabe K, Ohnuki M, Narita M, Ichisaka T, Tomoda K, et al. Induction of pluripotent stem cells from adult human fibroblasts by defined factors. Cell. 2007;131(5):861–72. 10.1016/j.cell.2007.11.019. 18035408 10.1016/j.cell.2007.11.019

[CR210] Takeda N, Jain R, LeBoeuf MR, Wang Q, Lu MM, Epstein JA. Interconversion between intestinal stem cell populations in distinct niches. Science. 2011;334(6061):1420–4. 10.1126/science.1213214. 22075725 10.1126/science.1213214 PMC3705713

[CR211] Tam PP, Behringer RR. Mouse gastrulation: the formation of a mammalian body plan. Mech Dev. 1997;68(1–2):3–25. 10.1016/s0925-4773(97)00123-8. 9431800 10.1016/s0925-4773(97)00123-8

[CR212] Tang B, Yoo N, Vu M, Mamura M, Nam JS, Ooshima A, et al. Transforming growth factor-beta can suppress tumorigenesis through effects on the putative cancer stem or early progenitor cell and committed progeny in a breast cancer xenograft model. Cancer Res. 2007;67(18):8643–52. 10.1158/0008-5472.Can-07-0982. 17875704 10.1158/0008-5472.CAN-07-0982 PMC2427144

[CR213] Thangaraj JL, Coffey M, Lopez E, Kaufman DS. Disruption of TGF-β signaling pathway is required to mediate effective killing of hepatocellular carcinoma by human iPSC-derived NK cells. Cell Stem Cell. 2024.10.1016/j.stem.2024.06.009. 10.1016/j.stem.2024.06.009 PMC11380586 38986609

[CR214] Theunissen TW, Powell BE, Wang H, Mitalipova M, Faddah DA, Reddy J, et al. Systematic identification of culture conditions for induction and maintenance of naive human pluripotency. Cell Stem Cell. 2014;15(4):471–87. 10.1016/j.stem.2014.07.002. 25090446 10.1016/j.stem.2014.07.002 PMC4184977

[CR215] Thomson JA, Itskovitz-Eldor J, Shapiro SS, Waknitz MA, Swiergiel JJ, Marshall VS, et al. Embryonic stem cell lines derived from human blastocysts. Science. 1998;282(5391):1145–7. 10.1126/science.282.5391.1145. 9804556 10.1126/science.282.5391.1145

[CR216] Tiedemann H, Asashima M, Grunz H, Knöchel W. Pluripotent cells (stem cells) and their determination and differentiation in early vertebrate embryogenesis. Dev Growth Differ. 2001;43(5):469–502. 10.1046/j.1440-169x.2001.00599.x. 11576166 10.1046/j.1440-169x.2001.00599.x

[CR217] Tseng YH, Kokkotou E, Schulz TJ, Huang TL, Winnay JN, Taniguchi CM, et al. New role of bone morphogenetic protein 7 in brown adipogenesis and energy expenditure. Nature. 2008;454(7207):1000–4. 10.1038/nature07221. 18719589 10.1038/nature07221 PMC2745972

[CR218] Vallier L, Mendjan S, Brown S, Chng Z, Teo A, Smithers LE, et al. Activin/Nodal signalling maintains pluripotency by controlling Nanog expression. Development. 2009;136(8):1339–49. 10.1242/dev.033951. 19279133 10.1242/dev.033951 PMC2687465

[CR219] van der Flier LG, Clevers H. Stem cells, self-renewal, and differentiation in the intestinal epithelium. Annu Rev Physiol. 2009;71:241–60. 10.1146/annurev.physiol.010908.163145. 18808327 10.1146/annurev.physiol.010908.163145

[CR220] von Bernhardi R, Cornejo F, Parada GE, Eugenín J. Role of TGFβ signaling in the pathogenesis of Alzheimer’s disease. Front Cell Neurosci. 2015;9:426. 10.3389/fncel.2015.00426. 26578886 10.3389/fncel.2015.00426 PMC4623426

[CR221] Voog J, Jones DL. Stem cells and the niche: a dynamic duo. Cell Stem Cell. 2010;6(2):103–15. 10.1016/j.stem.2010.01.011. 20144784 10.1016/j.stem.2010.01.011 PMC3012646

[CR222] Wang LD, Wagers AJ. Dynamic niches in the origination and differentiation of haematopoietic stem cells. Nat Rev Mol Cell Biol. 2011;12(10):643–55. 10.1038/nrm3184. 21886187 10.1038/nrm3184 PMC4040463

[CR223] Wang L, Li X, Huang W, Zhou T, Wang H, Lin A, et al. TGFβ signaling regulates the choice between pluripotent and neural fates during reprogramming of human urine derived cells. Sci Rep. 2016a;6:22484. 10.1038/srep22484. 26935433 10.1038/srep22484 PMC4776143

[CR224] Wang XH, Liu MN, Sun X, Xu CH, Liu J, Chen J, et al. TGF-β1 pathway affects the protein expression of many signaling pathways, markers of liver cancer stem cells, cytokeratins, and TERT in liver cancer HepG2 cells. Tumour Biol. 2016b;37(3):3675–81. 10.1007/s13277-015-4101-z. 26462837 10.1007/s13277-015-4101-z

[CR225] Wang H, Wang M, Wang Y, Wen Y, Chen X, Wu D, et al. MSX2 suppression through inhibition of TGFβ signaling enhances hematopoietic differentiation of human embryonic stem cells. Stem Cell Res Ther. 2020;11(1):147. 10.1186/s13287-020-01653-3. 32248833 10.1186/s13287-020-01653-3 PMC7132876

[CR226] Wang B, Wang Y, Chen H, Yao S, Lai X, Qiu Y, et al. Inhibition of TGFβ improves hematopoietic stem cell niche and ameliorates cancer-related anemia. Stem Cell Res Ther. 2021;12(1):65. 10.1186/s13287-020-02120-9. 33461597 10.1186/s13287-020-02120-9 PMC7814632

[CR227] Wang X, Song C, Ye Y, Gu Y, Li X, Chen P, et al. BRD9-mediated control of the TGF-β/Activin/Nodal pathway regulates self-renewal and differentiation of human embryonic stem cells and progression of cancer cells. Nucleic Acids Res. 2023;51(21):11634–51. 10.1093/nar/gkad907. 37870468 10.1093/nar/gkad907 PMC10681724

[CR228] Wang Q, Xiong F, Wu G, Wang D, Liu W, Chen J, et al. SMAD proteins in TGF-β signalling pathway in cancer: regulatory mechanisms and clinical applications. Diagnostics (Basel). 2023;13(17):2769. 10.3390/diagnostics13172769. 37685308 10.3390/diagnostics13172769 PMC10487229

[CR229] Warner K, Luther C, Takei F. Lymphoid progenitors in normal mouse lymph nodes develop into NK cells and T cells in vitro and in vivo. Exp Hematol. 2012;40(5):401–6. 10.1016/j.exphem.2012.01.009. 22269116 10.1016/j.exphem.2012.01.009

[CR230] Watabe T, Miyazono K. Roles of TGF-beta family signaling in stem cell renewal and differentiation. Cell Res. 2009;19(1):103–15. 10.1038/cr.2008.323. 19114993 10.1038/cr.2008.323

[CR231] Watanabe M, Buth JE, Haney JR, Vishlaghi N, Turcios F, Elahi LS, et al. TGFβ superfamily signaling regulates the state of human stem cell pluripotency and capacity to create well-structured telencephalic organoids. Stem Cell Reports. 2022;17(10):2220–38. 10.1016/j.stemcr.2022.08.013. 10.1016/j.stemcr.2022.08.013 PMC9561534 36179695

[CR232] Williams JT, Southerland SS, Souza J, Calcutt AF, Cartledge RG. Cells isolated from adult human skeletal muscle capable of differentiating into multiple mesodermal phenotypes. Am Surg. 1999;65(1):22–6. 10.1177/000313489906500106. 9915526

[CR233] Woosley AN, Dalton AC, Hussey GS, Howley BV, Mohanty BK, Grelet S, et al. TGFβ promotes breast cancer stem cell self-renewal through an ILEI/LIFR signaling axis. Oncogene. 2019;38(20):3794–811. 10.1038/s41388-019-0703-z. 30692635 10.1038/s41388-019-0703-z PMC6525020

[CR234] Wrana JL. Regulation of Smad Activity. Cell. 2000;100(2):189–92. 10.1016/S0092-8674(00)81556-1. 10660041 10.1016/s0092-8674(00)81556-1

[CR235] Wrana JL. The Secret Life of Smad4. Cell. 2009;136(1):13–4. 10.1016/j.cell.2008.12.028. 19135880 10.1016/j.cell.2008.12.028

[CR236] Wu X, Shen Q, Zhang Z, Zhang D, Gu Y, Xing D. Photoactivation of TGFβ/SMAD signaling pathway ameliorates adult hippocampal neurogenesis in Alzheimer’s disease model. Stem Cell Res Ther. 2021;12(1):345. 10.1186/s13287-021-02399-2. 34116709 10.1186/s13287-021-02399-2 PMC8196501

[CR237] Wu JY, Yeager K, Tavakol DN, Morsink M, Wang B, Soni RK, et al. Directed differentiation of human iPSCs into mesenchymal lineages by optogenetic control of TGF-β signaling. Cell Rep. 2023;42(5): 112509. 10.1016/j.celrep.2023.112509. 37178118 10.1016/j.celrep.2023.112509 PMC10278972

[CR238] Xiao L, Yuan X, Sharkis SJ. Activin A maintains self-renewal and regulates fibroblast growth factor, Wnt, and bone morphogenic protein pathways in human embryonic stem cells. Stem Cells. 2006;24(6):1476–86. 10.1634/stemcells.2005-0299. 16456129 10.1634/stemcells.2005-0299

[CR239] Xu RH, Sampsell-Barron TL, Gu F, Root S, Peck RM, Pan G, et al. NANOG is a direct target of TGFbeta/activin-mediated SMAD signaling in human ESCs. Cell Stem Cell. 2008;3(2):196–206. 10.1016/j.stem.2008.07.001. 18682241 10.1016/j.stem.2008.07.001 PMC2758041

[CR240] Xu X, Zheng L, Yuan Q, Zhen G, Crane JL, Zhou X, et al. Transforming growth factor-β in stem cells and tissue homeostasis. Bone Res. 2018;6:2. 10.1038/s41413-017-0005-4. 29423331 10.1038/s41413-017-0005-4 PMC5802812

[CR241] Xu Y, Zhao J, Ren Y, Wang X, Lyu Y, Xie B, et al. Derivation of totipotent-like stem cells with blastocyst-like structure forming potential. Cell Res. 2022;32(6):513–29. 10.1038/s41422-022-00668-0. 35508506 10.1038/s41422-022-00668-0 PMC9160264

[CR242] Yamashita M, Aoki H, Hashita T, Iwao T, Matsunaga T. Inhibition of transforming growth factor beta signaling pathway promotes differentiation of human induced pluripotent stem cell-derived brain microvascular endothelial-like cells. Fluids Barriers CNS. 2020;17(1):36. 10.1186/s12987-020-00197-1. 32456699 10.1186/s12987-020-00197-1 PMC7249446

[CR243] Yamazaki S, Iwama A, Takayanagi S, Eto K, Ema H, Nakauchi H. TGF-beta as a candidate bone marrow niche signal to induce hematopoietic stem cell hibernation. Blood. 2009;113(6):1250–6. 10.1182/blood-2008-04-146480. 18945958 10.1182/blood-2008-04-146480

[CR244] Yamazaki S, Ema H, Karlsson G, Yamaguchi T, Miyoshi H, Shioda S, et al. Nonmyelinating Schwann cells maintain hematopoietic stem cell hibernation in the bone marrow niche. Cell. 2011;147(5):1146–58. 10.1016/j.cell.2011.09.053. 22118468 10.1016/j.cell.2011.09.053

[CR245] Yang L, Chang N, Liu X, Han Z, Zhu T, Li C, et al. Bone marrow-derived mesenchymal stem cells differentiate to hepatic myofibroblasts by transforming growth factor-β1 via sphingosine kinase/sphingosine 1-phosphate (S1P)/S1P receptor axis. Am J Pathol. 2012;181(1):85–97. 10.1016/j.ajpath.2012.03.014. 22609227 10.1016/j.ajpath.2012.03.014

[CR246] Ying QL, Nichols J, Chambers I, Smith A. BMP induction of Id proteins suppresses differentiation and sustains embryonic stem cell self-renewal in collaboration with STAT3. Cell. 2003;115(3):281–92. 10.1016/s0092-8674(03)00847-x. 14636556 10.1016/s0092-8674(03)00847-x

[CR247] Yu ZX, Li PY, Li K, Miao SY, Wang LF, Song W. Progress on spermatogonial stem cell microenvironment. Yi Chuan. 2022;44(12):1103–16. 10.16288/j.yczz.22-136. 36927556 10.16288/j.yczz.22-136

[CR248] Yue D, Zhang Z, Li J, Chen X, Ping Y, Liu S, et al. Transforming growth factor-beta1 promotes the migration and invasion of sphere-forming stem-like cell subpopulations in esophageal cancer. Exp Cell Res. 2015;336(1):141–9. 10.1016/j.yexcr.2015.06.007. 26096658 10.1016/j.yexcr.2015.06.007

[CR249] Zakrzewski W, Dobrzyński M, Szymonowicz M, Rybak Z. Stem cells: past, present, and future. Stem Cell Res Ther. 2019;10(1):68. 10.1186/s13287-019-1165-5. 30808416 10.1186/s13287-019-1165-5 PMC6390367

[CR250] Zamani N, Brown CW. Emerging roles for the transforming growth factor-{beta} superfamily in regulating adiposity and energy expenditure. Endocr Rev. 2011;32(3):387–403. 10.1210/er.2010-0018. 21173384 10.1210/er.2010-0018 PMC3365795

[CR251] Zhang J, He XC, Tong WG, Johnson T, Wiedemann LM, Mishina Y, et al. Bone morphogenetic protein signaling inhibits hair follicle anagen induction by restricting epithelial stem/progenitor cell activation and expansion. Stem Cells. 2006;24(12):2826–39. 10.1634/stemcells.2005-0544. 16960130 10.1634/stemcells.2005-0544

[CR252] Zhou J, Su P, Li D, Tsang S, Duan E, Wang F. High-efficiency induction of neural conversion in human ESCs and human induced pluripotent stem cells with a single chemical inhibitor of transforming growth factor beta superfamily receptors. Stem Cells. 2010;28(10):1741–50. 10.1002/stem.504. 20734356 10.1002/stem.504 PMC3322377

[CR253] Zubeldia IG, Bleau AM, Redrado M, Serrano D, Agliano A, Gil-Puig C, et al. Epithelial to mesenchymal transition and cancer stem cell phenotypes leading to liver metastasis are abrogated by the novel TGFβ1-targeting peptides P17 and P144. Exp Cell Res. 2013;319(3):12–22. 10.1016/j.yexcr.2012.11.004. 10.1016/j.yexcr.2012.11.004 23153552

[CR254] Zuk PA, Zhu M, Ashjian P, De Ugarte DA, Huang JI, Mizuno H, et al. Human adipose tissue is a source of multipotent stem cells. Mol Biol Cell. 2002;13(12):4279–95. 10.1091/mbc.e02-02-0105. 12475952 10.1091/mbc.E02-02-0105 PMC138633

